# Determination of the Chemical Structures of Tandyukisins B–D, Isolated from a Marine Sponge-Derived Fungus

**DOI:** 10.3390/md13053231

**Published:** 2015-05-21

**Authors:** Takeshi Yamada, Yoshihide Umebayashi, Maiko Kawashima, Yuma Sugiura, Takashi Kikuchi, Reiko Tanaka

**Affiliations:** Osaka University of Pharmaceutical Sciences, 4-20-1, Nasahara, Takatsuki, Osaka 569-1094, Japan; E-Mails: j.apricot_woods-0621@docomo.ne.jp (Y.U.); when-you.with-upon.a-star@disney.ne.jp (M.K.); ye9m13bxwt2kkbzpyxyf@docomo.ne.jp (Y.S.); t.kikuchi@gly.oups.ac.jp (T.K.); tanakar@gly.oups.ac.jp (R.T.)

**Keywords:** decalin, *Trichoderma harzianum*, marine microorganism, *Halichondria okadai*, cytotoxicity

## Abstract

Tandyukisins B–D (**1**–**3**), novel decalin derivatives, have been isolated from a strain of *Trichoderma harzianum* OUPS-111D-4 originally derived from the marine sponge *Halichondria okadai*, and their structures have been elucidated on the basis of spectroscopic analyses using 1D and 2D NMR techniques. In addition, their chemical structures were established by chemical transformation. They exhibited weak cytotoxicity, but selective growth inhibition on panel screening using 39 human cancer cell lines.

## 1. Introduction

**Figure 1:**
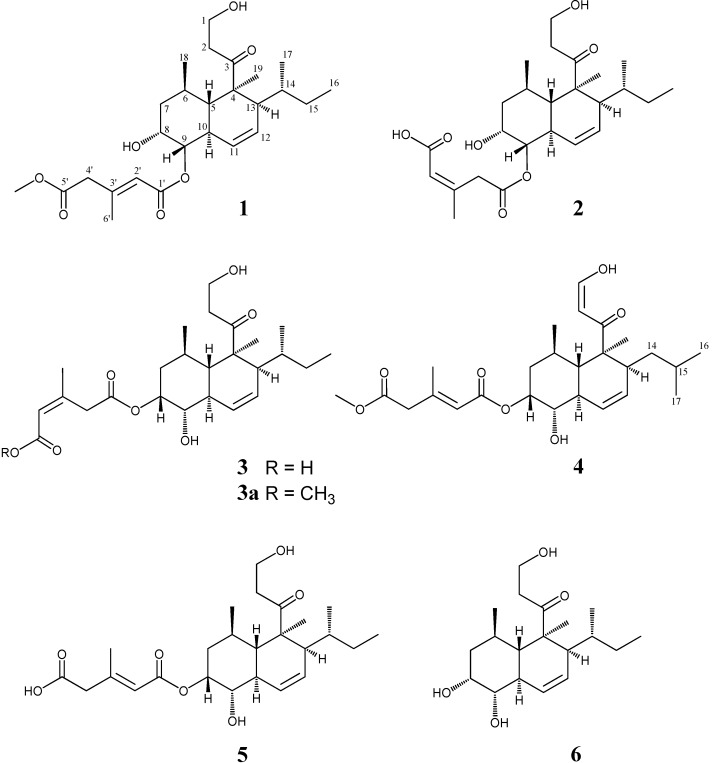
Structures of metabolites **1**–**6**.

Marine microorganisms are potentially prolific sources of highly bioactive secondary metabolites that may offer useful leads in the development of new pharmaceutical agents. Based on the fact that some of the bioactive materials isolated from marine animals have been produced by associated microorganisms, we have focused our attention on new antitumor materials from microorganisms isolated from marine organisms [1,2,3]. We previously reported that a cytotoxic compound, tandyukisin A (**4**), a novel decalin derivative with an enolic β-ketoaldehyde, was isolated from a strain of *Trichoderma harzianum* OUPS-111D-4 originally derived from the marine sponge *Halichondria okadai* [4]. In addition, the known compounds trichoharzin (**5**) and eujavanicol A (**6**) were isolated together with it. Our continuing search for cytotoxic metabolites from this strain led to the isolation of three new decalin derivatives designated tandyukisins B–D (**1**–**3**) (Figure 1). These metabolites exhibited moderate cytotoxicity against a disease-oriented panel of 39 human cancer cell lines (HCC panel); however, they exhibited selective growth inhibition against central nervous system tumor cell lines. Trichoharzin [5] isolated from the same class of fungus, eujavanicols [6,7] from *Eupenicillium javanicum*, betaenones [8] from *Phoma betae*, stemphyloxin I [9] from *Stemphylium botryosum*, fusarielins [10] from *Fusarium* sp., pannomycin [11] from *Geomyces pannorum*, and australifungin [12] from *Spopormiella australis* have been reported as metabolites with a similar alkylated decalin skeleton. They have various bioactivities, such as antifungal, phytotoxic, and antibacterial; however, cytotoxicities of these compounds have not been reported to date. We describe herein the absolute stereostructures and biological activities of **1**–**3**.

## 2. Results and Discussion

*T. harzianum*, a microorganism from *H. okadai*, was cultured at 27 °C for six weeks in a medium (70 L) containing 1% glucose, 1% malt extract and 0.05% peptone in artificial seawater adjusted to pH 7.5. After incubation, the EtOAc extract of the culture filtrate was purified employing a stepwise combination of silica gel column chromatography and reverse phase HPLC to afford tandyukisins B (**1**) (3.8 mg), C (**2**) (8.0 mg), and D (**3**) (12.0 mg) as pale yellow oil, respectively.

**Figure 2:**
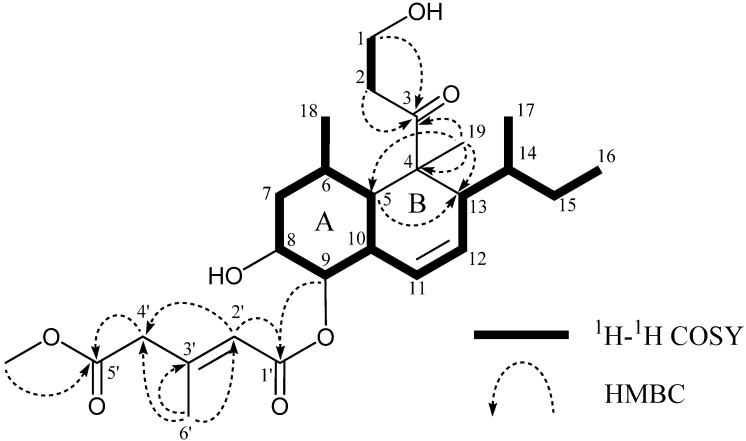
Selected ^1^H–^1^H COSY and HMBC correlations of **1**.

**Figure 3:**
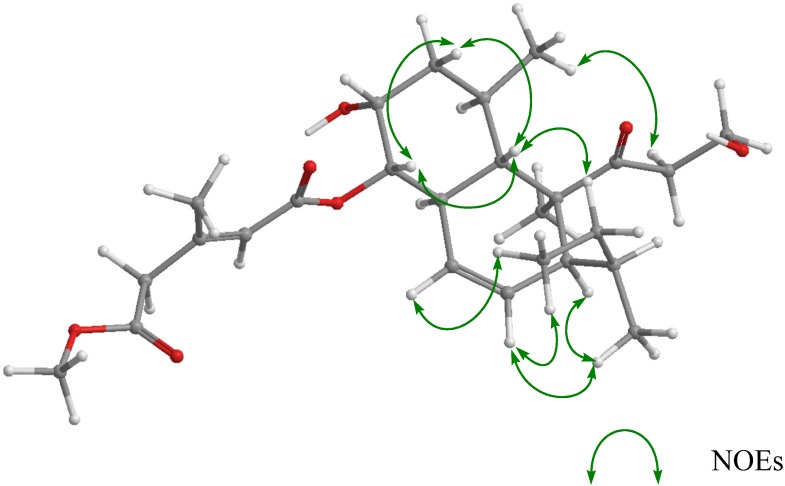
Key NOESY correlations of **1**.

Tandyukisin B (**1**) had the molecular formula C_2__6_H_40_O_4_ as established from the [M + H]^+^ peak in HR FAB-MS. Its IR spectrum exhibited bands at 2958, 1715 and 1651 cm^−1^, characteristic of hydroxy groups, esters, and ketone. Close inspection of the ^1^H and ^13^C NMR spectra (Table 1) of **1** using DEPT and ^1^H-^13^C correlation spectroscopy (HSQC) revealed the presence of one primary methyl (C-16), two secondary methyls (C-17 and C-18), one tertiary methyl (C-19), one olefin methyl (C-6′), one methoxy group (5′-OCH_3_), five sp^3^-hybridized methylenes (C-1, C-2, C-7, C-15, and C-4′) including one oxygen-bearing carbon (C-1), six oxygen-bearing sp^3^-methines (C-5, C-6, C-8, C-9, C-10, C-13, and C-14) including two oxygen-bearing carbons (C-8 and C-9), three sp^2^-methines (C-11, C-12, and C-2′), one quaternary sp^3^-carbon (C-4), one quaternary sp^2^-carbon (C-3′), three carbonyl groups (C-3, C-1′, and C-5′) including two ester carbonyls (C-1′ and C-5′). ^1^H–^1^H COSY analysis of **1** gave two partial structural units, as shown by the bold lines in Figure 2. The connection of these units with the remaining functional groups was determined on the basis of HMBC correlations, summarized in Figure 2, and the planar structure of **1** was elucidated, as shown in Figure 2. **1** was markedly different from **4** and **5** regarding the position of the esterified hydroxyl group. NOE correlation between H-2′ and H-4′ (Figure 3) revealed that **1** had the same geometrical configuration in the side chain as those of **4** and **5**.

**Table 1:** ^1^H and ^13^C NMR Spectral Data for **1**, **2**, **3**, and **5**.

Position	1					2				3					5			
	δ_H_^a^			δ_C_		δ_H_^a^		δ_C_		δ_H_^a^			δ_C_		δ_H_^a^		δ_C_	
1A	3.83		ddd	58.0	(t)	3.84	ddd	58.0	(t)	3.83		ddd	58.0	(t)	3.84	ddd	58.0	(t)
1B	3.89		ddd			3.90	ddd			3.91		ddd			3.90	ddd		
2A	2.66		ddd	41.2	(t)	2.67	ddd	41.1	(t)	2.67		ddd	41.2	(t)	2.67	ddd	41.2	(t)
2B	2.87		ddd			2.85	ddd			2.86		ddd			2.86	ddd		
3				215.2	(s)			215.2	(s)				215.2	(s)			215.5	(s)
4				52.5	(s)			52.5	(s)				52.5	(s)			52.5	(s)
5	2.06		t	43.6	(d)	2.03	t	43.4	(d)	1.96		t	43.1	(d)	1.98	t	43.0	(d)
6	1.82		m	30.3	(d)	1.73	m	30.5	(d)	1.59		m	31.4	(d)	1.62	m	31.5	(d)
7α	1.86		dt	40.7	(t)	1.83	dt	40.1	(t)	1.83		dt	39.1	(t)	1.87	dt	39.0	(t)
7β	1.55		td			1.53	td			1.55		td			1.56	td		
8	4.13		q	67.6	(d)	4.28	q	66.7	(d)	5.26		q	73.3	(d)	5.26	q	72.7	(d)
9	4.78		dd	77.4	(d)	4.55	dd	78.8	(d)	3.48		dd	74.2	(d)	3.56	dd	74.4	(d)
10	2.46		tdd	36.2	(d)	2.47	brt	36.0	(d)	2.08		tdd	40.4	(d)	2.12	tdd	40.3	(d)
11	5.62		brd	125.0	(d)	5.69	drd	125.1	(d)	6.06		dt	125.9	(d)	6.04	brd	125.8	(d)
12	5.74		ddd	124.5	(d)	5.67	dd	124.5	(d)	5.69		ddd	123.7	(d)	5.70	ddd	123.8	(d)
13	1.94		m	52.4	(d)	1.94	m	52.3	(d)	1.94		m	52.4	(d)	1.94	m	52.4	(d)
14		1.12	m	37.2	(d)	1.12	m	37.2	(d)		1.12	m	37.2	(d)	1.12	m	37.2	(d)
15A		0.74	m	24.4	(t)	0.72	m	24.5	(t)		0.74	m	24.4	(t)	0.74	m	24.4	(t)
15B		1.50	m			1.47	m				1.47	m			1.47	m		
16		0.76	t	12.5	(q)	0.75	t	12.5	(q)		0.76	t	12.5	(q)	0.76	t	12.5	(q)
17		0.92	d	19.1	(q)	0.92	d	19.2	(q)		0.93	d	19.2	(q)	0.93	d	19.3	(q)
18		0.60	d	22.3	(q)	0.59	d	22.3	(q)		0.59	d	22.3	(q)	0.59	d	22.2	(q)
19		1.26	s	19.4	(q)	1.26	s	19.3	(q)		1.25	s	19.3	(q)	1.26	s	19.4	(q)
1′				164.9	(s)			170.0	(s)				168.9	(s)			166.2	(s)
2′A		5.88	s	118.9	(d)	3.29	d	39.7	(t)		5.92	s	118.1	(d)	5.88	s	119.7	(d)
2′B						3.77	d											
3′				152.0	(s)			153.4	(s)				153.9	(s)			151.7	(s)
4′A		3.19	s	45.7	(t)	5.94	s	118.5	(d)		3.54	d	39.6	(t)	3.19	s	45.4	(t)
4′B											3.73	d						
5′				170.2	(s)			168.9	(s)				169.8	(s)			173.5	(s)
6′		2.27	s	19.2	(q)	2.08	s	27.4	(q)		2.05	s	26.9	(q)	2.27	s	19.1	(q)
5′-OCH_3_		3.73	s	52.2	(q)													

^a^ ^1^H chemical shift values (d ppm from SiMe4) followed by multiplicity.

The relative stereochemistry of **1** was deduced from NOESY experiments (Figure 3). NOE correlations (H-5/H-7β, H-5/H-9, H-9/H-7β, and H-6/H-10) suggested that the A ring existed in a chair conformation with H-5, H-7β, and H-9 in coaxial arrangements. This evidence and the vicinal coupling constants (*J*_7α,8_ = *J*_7β,8_ = *J*_9,8_ = 2.5 Hz) showed that the esterified side chain at C-9 was oriented *cis* to 8-OH in an equatorial arrangement. NOE correlations (H-10/H-19, H-5/H-15, and H-2B/H-18) in the B ring revealed that it existed in a half chair conformation, and the *sec*-butyl group was oriented *trans* to C-19. For the stereochemistry at C-14 in the *sec*-butyl group, the observed NOE correlations in **1** were equal to those of **5** [5], which revealed the stereochemistry at C-14, *i.e.*, NOE correlations (H-5/H-15B, H-2A/H-13, H-2A/H-14, H-13/H-17, H-12/H-17, and H-12/H-16) suggested that the rotation of the *sec*-butyl group in its pseudo-axial arrangement was limited; therefore, the relative configuration for C-14 was deduced as S* (Figure 3). For the determination of the absolute stereostructure of **1**, alkali-hydrolysis was carried out. The treatment of **1** with NaOH in aqueous MeOH gave a hydrolysis product, which was identical to **6** in terms of ^1^H NMR data and the specific rotation (the hydrolysis product; [α]_D_ +46.5, **6**: [α]_D_ +41.1). This evidence led to the absolute stereostructure of **1**, and confirmed the stereochemistry at C-14 derived from NOE correlations.

Tandyukisin C (**2**) was assigned the same molecular formula C_25_H_38_O_7_ as **5** based on HR FAB-MS data. The general features of its UV, IR, and NMR spectra (Table 1) closely resembled those of **5** except for some of the ^1^H and ^13^C NMR signals (H-8 (Δδ_H_ 0.98), H-9 (Δδ_H_ 1.00), H-10 (Δδ_H_ 0.35), H-11 (Δδ_H_ 0.35), C-8 (Δδ_C_ 6.0), C-9 (Δδ_C_ 4.4), and C-10 (Δδ_C_ 4.2)). At this point, we thought that **2** also had an esterified side chain at C-9 as **1**. However, additional analysis of the NMR chemical shifts at the side chain of **2** showed marked differences from those of **5**, and suggested that its structure must be markedly different. ^1^H-^1^H COSY and HMBC experiments led to the elucidation of the planar structure of **2**, which was the same as those of **1** and **5** except for the esterified side chain C-1′–C-6′. In the HMBC experiments, the correlations (H-9/C-1′, H-2′/C-1′, H-2′/C-4′, H-4′/C-5′, H-6′/C-2′, H-6′/C-3′, and H-6′/C-4′) demonstrated that another carboxylic acid in the side chain, which was dicarboxylic acid originally, condensed to the hydroxyl group at C-9. In addition, a detailed examination of NOESY led to the finding that the geometrical configuration in the side chain was *Z*, while those of **1** and **5** were *E*, *i.e.*, the NOE correlations between H-6′ and H-4′ in **2** were observed instead of those between H-2′ and H-4′ in **1** and **5**. For the stereochemistry of **2**, all NOE correlations except for the above were identical to those of **1** and **5**. In addition, alkali-hydrolysis in the same manner as described above gave a hydrolysis product, which was identical to **6** in terms of spectral data, including the specific rotation ([α]_D_ +42.7). The above evidence established the absolute stereostructure of **2** as shown in Figure 1.

Tandyukisin D (**3**) was assigned the same molecular formula C_25_H_38_O_7_ as **2**, based on deductions made from HR FAB-MS data. Its spectral data were similar to those of the related compounds **1**, **2**, **4**, and **5** isolated to date. Especially, there were marked similarities between **3** and **5** except for the NMR signals of the side chain. On the other hand, the same NMR chemical shifts for the side chain of **3** corresponded exactly to those of **2** (Table 1). Based on the ^1^H-^1^H COSY and HMBC correlations for **3**, the planar structure esterified at C-8 could be deduced together with the ^1^H NMR chemical shift at H-8 (δ_H_ 5.26); however, an HMBC correlation from H-8 to C-1′ was not observed. In order to examine which of the two carboxy groups in the side chain dicarboxylic acid condensed to 8-OH, we performed methylation of **3**. The addition of trimethylsilyldiazomethane to **3** gave methyl ester derivative **3a**. The HMBC correlation from the methyl group imported newly in **3a** assigned a carboxy group as C-5′, and then another one was assigned as C-1′ condensed to 8-OH. Consequently, the assignment of the NMR signals for the side chain was completed. In the NOESY experiment for **3**, the correlations between H-6′ and H-4′ showed that the geometry in the side chain was *Z*, as that of **2**. A detailed examination of NOESY revealed the relative configuration of the chiral centers, except for the above, which were identical to those of **1**, **2**, and **5**. The absolute stereostructure of **3** was confirmed by alkali-hydrolysis. As expected, the hydrolysis product was identical to **6** in terms of spectral data, including the specific rotation ([α]_D_ +43.8).

As primary screening for antitumor activity, cancer cell growth inhibitory properties of tandyukisins B–D (**1**–**3**) were examined using a disease-oriented panel of 39 human cell lines (HCC panel) [13,14]. The effective concentration (MG-MID), delta value, and range value for **1** did not show significant cytotoxic activity (effective value: MG-MID < −5, delta ≥ 0.5, and range ≥ 1.0) (Table 2). However, **1**–**3** exhibited slightly selective growth inhibition against the central nervous system cancer SNB-75 cell line in the HCC panel (log GI_50_ values: −4.36, −4.56, and −4.54, respectively).

**Table 2:** Cytotoxity of **1**–**3** against a panel of 39 human cancer cell lines.

Sample	1	2	3
MG-MID ^a^	−4.01	−4.04	−4.01
Delta ^b^	0.35	0.52	0.53
Range ^c^	0.36	0.56	0.54

^a^ Mean value of log GI_50_ over all cell lines tested; ^b^ The difference in log GI_50_ value of the most sensitive cell and MG-MID value; ^c^ The difference in log GI_50_ value of the most sensitive cell and the least sensitive cell.

## 3. Experimental Section

### 3.1. General Experimental Procedures

UV spectra were recorded on a Shimadzu spectro-photometer U-2000 and IR spectra on a JASCO FT/IR-680 Plus. NMR spectra were recorded at 27 °C on Agilent-NMR-vnmrs600 with tetramethylsilane (TMS) as an internal reference. Mass spectra were determined using a Hitachi M-4000H mass spectrometer. ORD were recorded on a JASCO J-820 polarimeters. Liquid chromatography over silica gel (mesh 230–400) was performed in a medium pressure. HPLC was run on a JASCO PU-1586 equipped with a differential refractometer (RI-1531) and Cosmosil Packed Column 5C_18_-MSII (25 cm × 20 mm i.d.). Analytical TLC was performed on precoated Merck aluminum sheets (DC-Alufolien Kieselgel 60 F254, 0.2 mm) with the solvent system CH_2_Cl_2_–MeOH (19:1), and compounds were viewed under UV lamp and sprayed with 10% H_2_SO_4_ followed by heating.

### 3.2. Fungal Material

The fungus *Trichoderma harzianum* was isolated from a piece of inner tissue of the marine sponge *Halichondria okadai* collected at collected in Osaka bay, Japan in October 2008. The fungal strain was identified by Techno Suruga Laboratory Co., Ltd. (Shizuoka, Japan). The sponge was wiped with EtOH and its snip applied to the surface of nutrient agar layered in a Petri dish. Serial transfers of one of the resulting colonies provided a pure strain of *T. harzianum*.

### 3.3. Culturing and Isolation of Metabolites

The fungal strain was cultured at 27 °C for six weeks in a liquid medium (70 L) containing 1% glucose, 1% malt extract and 0.05% pepton in artificial seawater adjusted to pH 7.5. The culture filtrate was extracted thrice with EtOAc. The combined extracts were evaporated *in vacuo* to afford a mixture of crude metabolites (17.2 g). The EtOAc extract was chromatographed on a silica gel column with a CHCl_3_–MeOH gradient as the eluent. The MeOH–CHCl_3_ (1:99) eluate (1.6 g) was repeated a silica gel column with a *n*-hexane–EtOAc gradient as the eluent. The *n*-hexane–EtOAc (30:70) eluate (323.2 mg) was purified by HPLC using MeOH–H_2_O (73:27) as the eluent to afford **1** (3.8 mg). In the second silica gel column chromatography, another *n*-hexane–EtOAc (30:70) eluate (292.6 mg) was purified by HPLC using MeOH–H_2_O (70:30) as the eluent to afford **2** (8.0 mg) and **3** (12.0 mg).

Tandyukisin B (**1**) Pale yellow oil; [α]_D_^22^ −27.9 (*c* 0.08, EtOH); UV λ_max_ (EtOH)/nm: 341 (log ε 2.14). IR (neat) ν_max_/cm^−1^: 2958, 1715, 1651. FABMS *m*/*z* (rel. int.): 487 ([M + Na]^+^, 11.3%) 465 ([M + H]^+^, 35.1%), 447 ([M − OH]^+^, 5.3%), 307 (77.1%), 289 (24.4%), 159 (26.8%), 141 (100%). HRFABMS *m*/*z* 465.2859 [M + H]^+^ (calcd for C_2__6_H_41_O_7_:465.2852). ^1^H and ^13^C NMR data are listed in Table 1 and Table S1 (SI).

Tandyukisin C (**2**) Pale yellow oil; [α]_D_^22^ +16.2 (*c* 0.10, EtOH); UV λ_max_ (EtOH)/nm: 341 (log ε 2.35). IR (neat) ν_max_/cm^−1^: 2960, 1698, 1652. FABMS *m*/*z* (rel. int.): 473 ([M + Na]^+^, 100%) 451 ([M + H]^+^, 28.2%), 433 ([M − OH]^+^, 32.9%), 307 (77.1%), 289 (60.2%), 159 (64.1%), 127 (77.9%). HRFABMS *m*/*z* 451.2703 [M + H]^+^ (calcd for C_2__5_H_39_O_7_:451.2696). ^1^H and ^13^C NMR data are listed in Table 1 and Table S2 (SI).

Tandyukisin D (**3**) Pale yellow oil; [α]_D_^22^ +32.2 (*c* 0.09, EtOH); UV λ_max_ (EtOH)/nm: 341 (log ε 2.72). IR (neat) ν_max_/cm^−1^: 2926, 1698. FABMS *m*/*z* (rel. int.): 473 ([M + Na]^+^, 7.8%) 451 ([M + H]^+^, 10.7%), 433 ([M − OH]^+^, 13.4%), 307 (16.6%), 289 (15.9%), 159 (20.7%), 154 (100%). HRFABMS *m*/*z* 451.2705 [M + H]^+^ (calcd for C_2__5_H_39_O_7_:451.2696). ^1^H and ^13^C NMR data are listed in Table 1 and Table S3 (SI).

### 3.4. Chemical Transformation

#### 3.4.1. The Hydrolysis of **1**–**3**

To a solution of **1** (2.2 mg) in MeOH (0.5 mL) 0.1 M NaOH aq. was added, and the reaction mixture was stirred at room temperature for 30 min. The reaction mixture extracted with diethyl ether thrice, and the organic layer was evaporated under reduced pressure. The residue was purified by HPLC using MeOH–H_2_O (70:30) as the eluent to afford **6** (1.0 mg). Using the same procedure, **2** (8.1 mg) and **3** (2.9 mg) were treated with 0.01 M NaOH aq. and purified by HPLC to afford **6** (1.2 mg and 0.9 mg), respectively.

#### 3.4.2. The Methylation of **3**

Trimethylsilyldiazomethane (2 mL) was added to a solution of **3** (1.4 mg) in CH_2_Cl_2_ (0.5 mL), and the reaction mixture was stirred at room temperature for 10 h. The reaction mixture was evaporated under reduced pressure. The residue (1.0 mg) was analyzed as **3a** without purification.

**3a** Pale yellow oil; FABMS *m*/*z* (rel. int.): 465 ([M + H]^+^, 16.1%), 447 ([M − OH]^+^, 23.0%), 141 (100%). HRFABMS *m*/*z* 465.2857 [M + H]^+^ (calcd for C_2__0_H_41_O_7_:465.2852). ^1^H and ^13^C NMR data are listed in Table S4 (SI).

## 4. Conclusions

In this study, three novel decalin derivatives with an enolic β-ketoaldehyde, tandyukisins B–D (**1**–**3**), were isolated from a strain of *Trichoderma harzianum* derived from the marine sponge. The chemical structures of these compounds were elucidated by spectral analyses and chemical transformation.

In the screening to search for seeds of antitumor agents, these metabolites did not exhibit significant cytotoxic activity in the HCC panel. However, we suggest that the low-level selectivity demonstrated by them may aid in the development of a new chemotherapeutical agent.

## Supplementary Files

Supplementary File 1
