# Supplementary material for: Determination of the Chemical Structures of Tandyukisins B–D, Isolated from a Marine Sponge-Derived Fungus
Source: Mar Drugs. 2015 May 21;13(5):3231–40. doi: 10.3390/md13053231 (PMC4446626; doi:10.3390/md13053231)
Supplement: Supplementary File 1 [file marinedrugs-13-03231-s001.pdf]

Supplementary Information

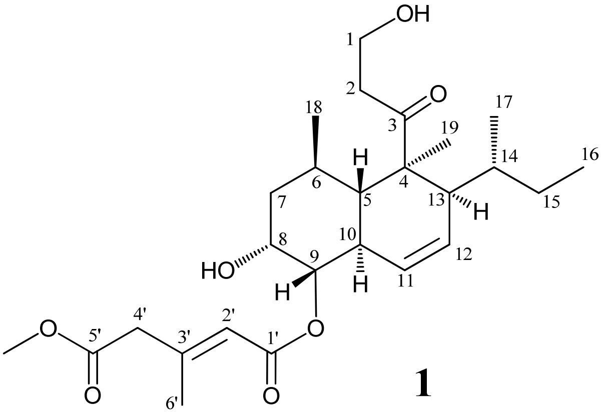

1

Figure S1. Compound 1.

Table S1. Spectral data including 2D NMR data for 1.

**Position**	**δ_H [a]**		***J*/Hz**	**^1H-^1H COSY**	**NOE [b]**	**δ_C**		**HMBC (C) [c]**
1A	3.83	ddd	11.5 (2B), 6.2 (2A), 3.5 (2B)	1B, 2A, 2B		58.0	(t)	3
1B	3.89	ddd	11.5 (1B), 6.2 (2B), 3.5 (2A)	1A, 2A, 2B	14			3
2A	2.66	ddd	18.5 (2B), 6.2 (1A), 3.5 (1B)	1A, 1B, 2B	13, 14, 19	41.2	(t)	1, 3
2B	2.87	ddd	18.5 (1B), 6.2 (1B), 3.5 (1A)	1A, 1B, 2A	18, 19			1, 3
3						215.2	(s)	
4						52.5	(s)	
5	2.06	t	11.5 (6, 10)	6, 10	7β, 9, 15B, 16, 18	43.6	(d)	3, 4, 6, 7, 9, 10, 13
6	1.82	m		5, 7β, 18	10, 19	30.3	(d)	
7α	1.86	dt	12.3 (7α), 2.5 (6, 8)	7β, 8	18	40.7	(t)	5, 8, 9
7β	1.55	td	12.3 (6, 7β), 2.5 (8)	6, 7α, 8	5, 9, 18			5, 6
8	4.13	q	2.5 (7α, 7β, 9)	7α, 7β, 9		67.6	(d)	6, 10
9	4.78	dd	11.5 (10), 2.5 (8)	8, 10	5, 7β, 11	77.4	(d)	1′, 10
10	2.46	tdd	11.5 (5, 9), 5.2 (11), 2.5 (12)	5, 9, 11, 12	6, 19	36.2	(d)	9, 11
11	5.62	br d	10.5 (12)	10, 12, 13	9, 16	125.0	(d)	5, 9, 10, 13
12	5.74	ddd	10.5 (11), 5.2 (13), 2.5 (10)	10, 11, 13	16, 17	124.5	(d)	10, 13
13	1.94	m		11, 12, 14	2A, 17, 19	52.4	(d)	19
14	1.12	m		13, 15A, 15B, 17	1B, 2A, 16	37.2	(t)	
15A	0.74	m		14		24.4	(d)	
15B	1.50	m		14, 16	5			13, 14, 16, 17
16	0.76	t	7.3 (15)	15B	5, 11, 12, 14, 17	12.5	(q)	14, 15
17	0.92	d	6.6 (14)	14	12, 13, 16	19.1	(q)	14, 15, 16
18	0.60	d	7.0 (6)	6	2B, 5, 7α, 7β	22.3	(q)	5, 6, 7
19	1.26	s			2A, 2B, 6, 10, 13	19.4	(q)	3, 4, 5, 13
1′						164.9	(s)	
2′	5.88	s			4′	118.9	(d)	1′, 3′, 4′, 6′
3′						152.0	(s)	
4′	3.19	s			2′, 6′	45.7	(t)	2′, 3′, 5′, 6′
5′						170.2	(s)	
6′	2.27	s			4′	19.2	(q)	2′, 3′, 4′
5′-OCH_3	3.73	s				52.2	(q)	5′

[a] ^1H chemical shift values (δppm from $SiMe_4$) followed by multiplicity and then the coupling constants (*J*/Hz). Figures in parentheses indicate the proton coupling with that position; [b] The correlations with geminal and vicinal protones are removed; [c] Long range ^1H-^{13}C correlations from H to C observed in the HMBC experiment.

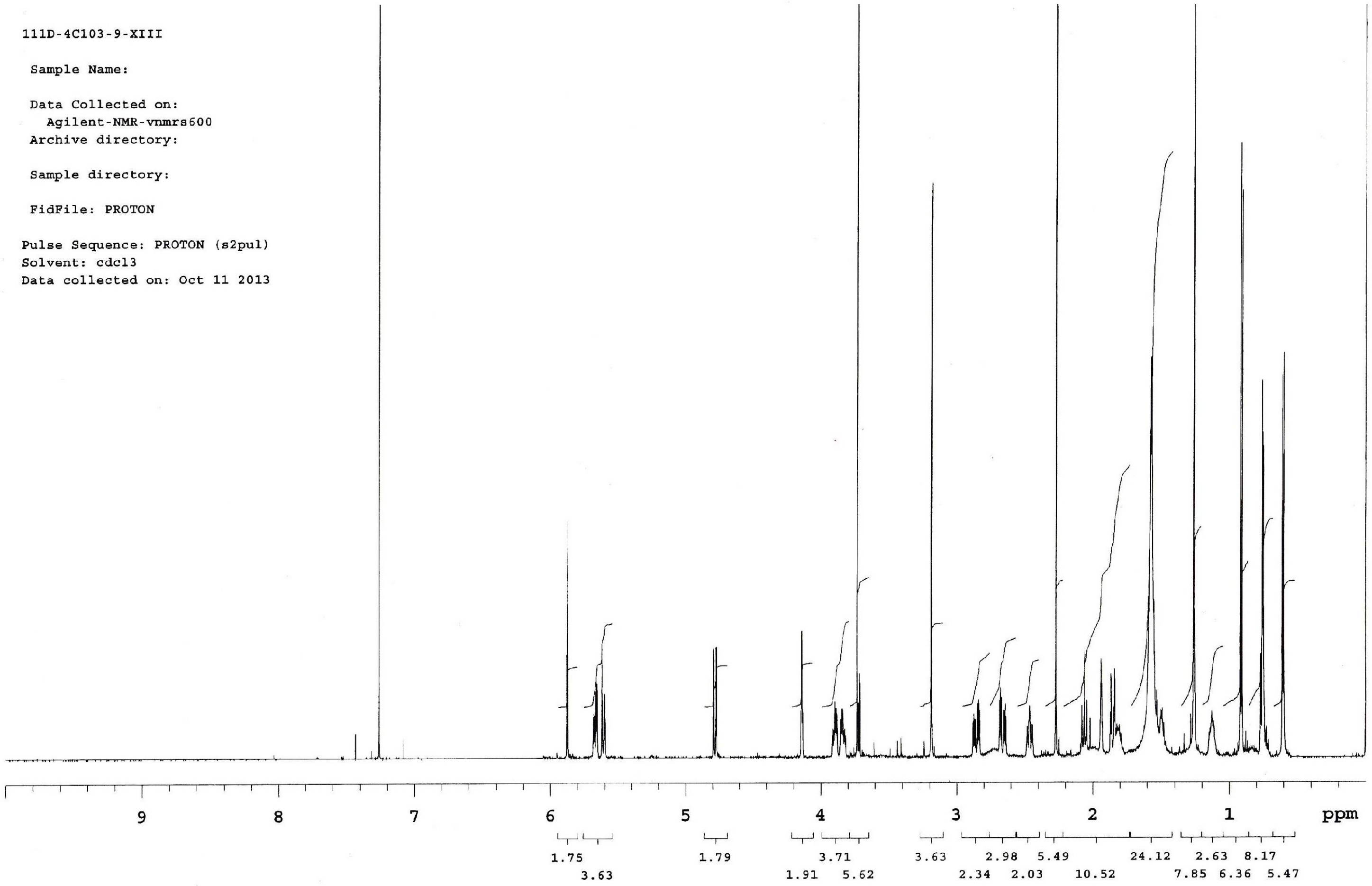

Figure S2. ^{1}H NMR spectrum of 1.

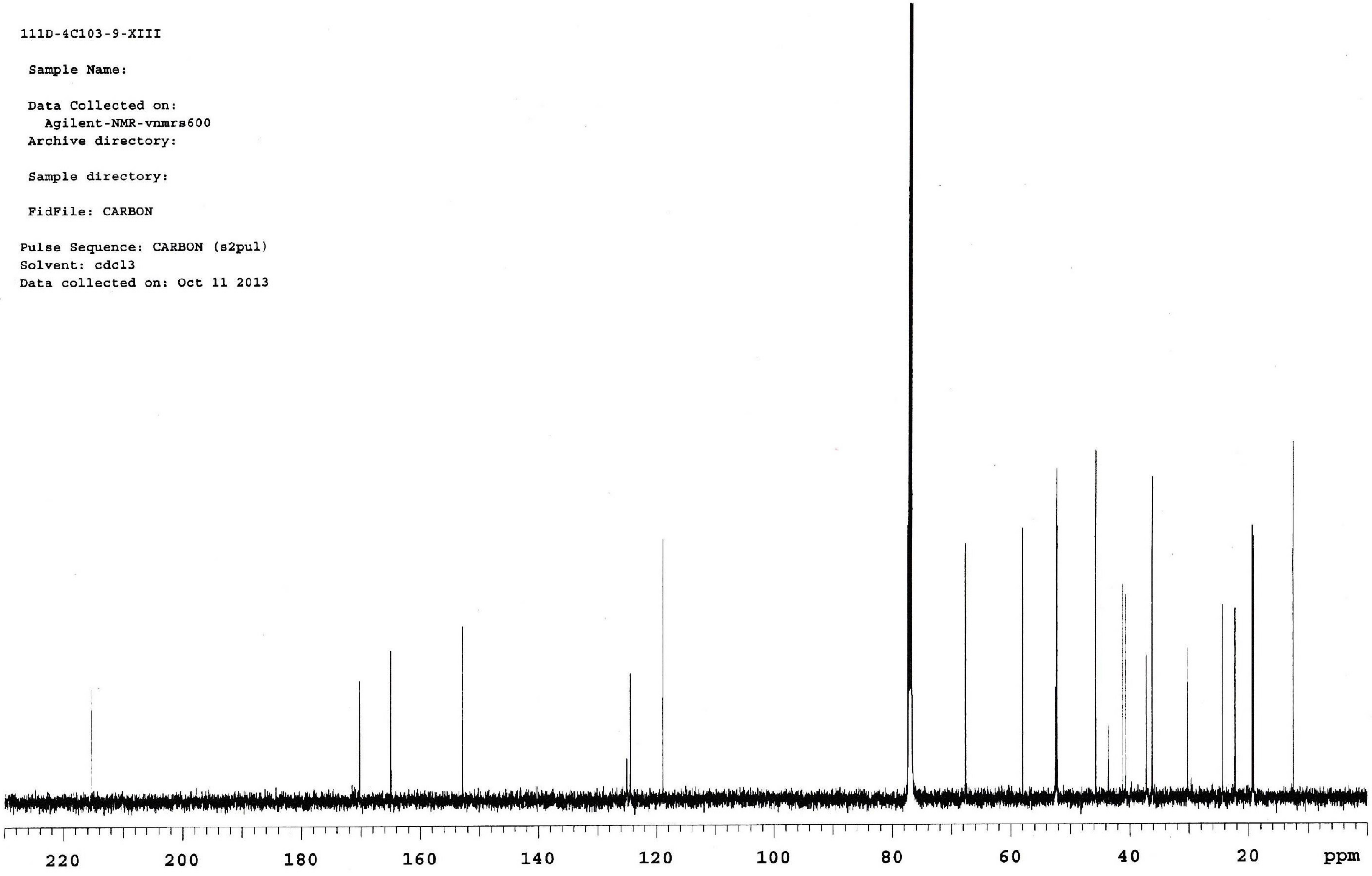

Figure S3. ^{13}C NMR spectrum of 1.

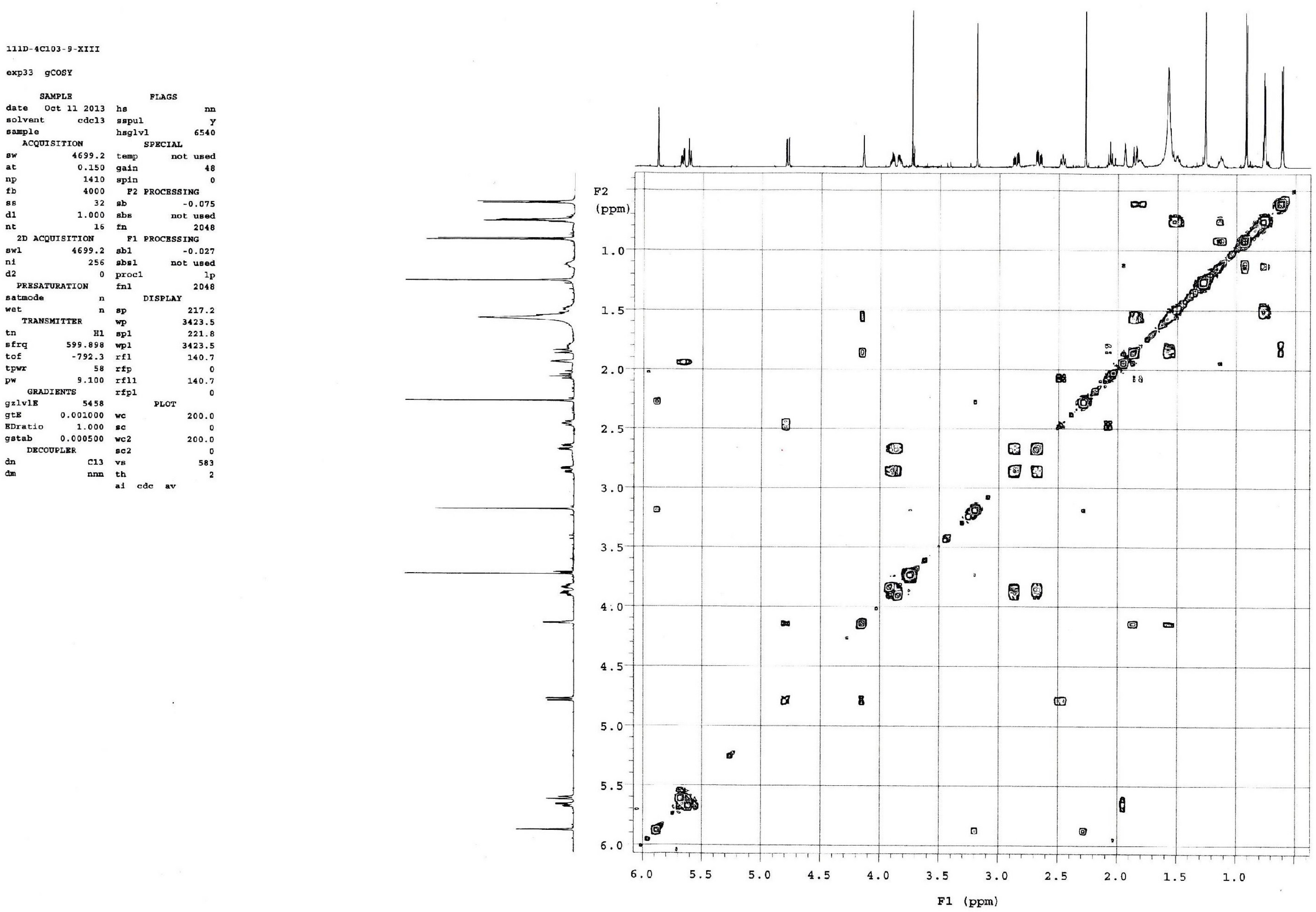

Figure S4. ^1H-^1H COSY of 1.

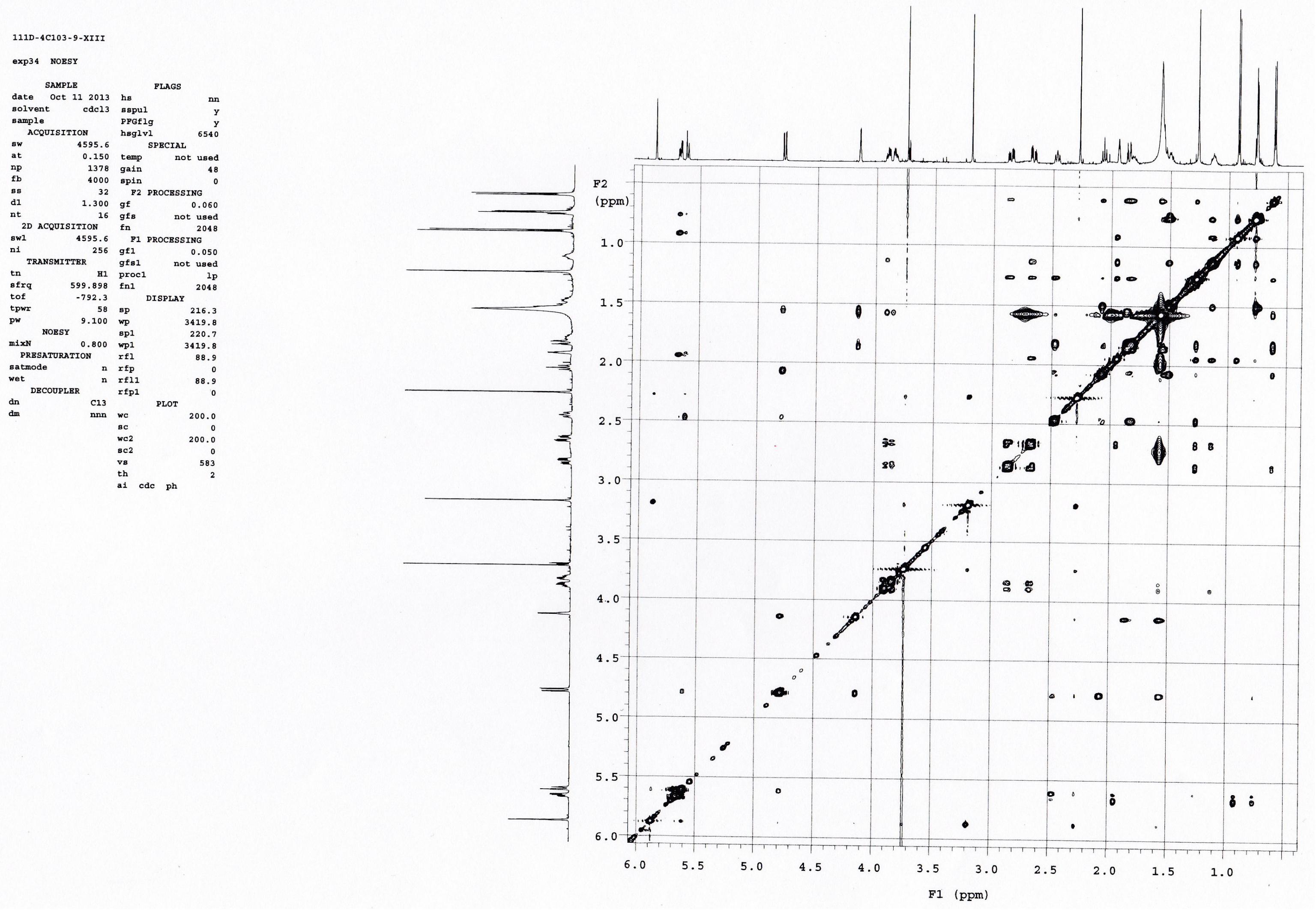

Figure S5. NOESY of 1.

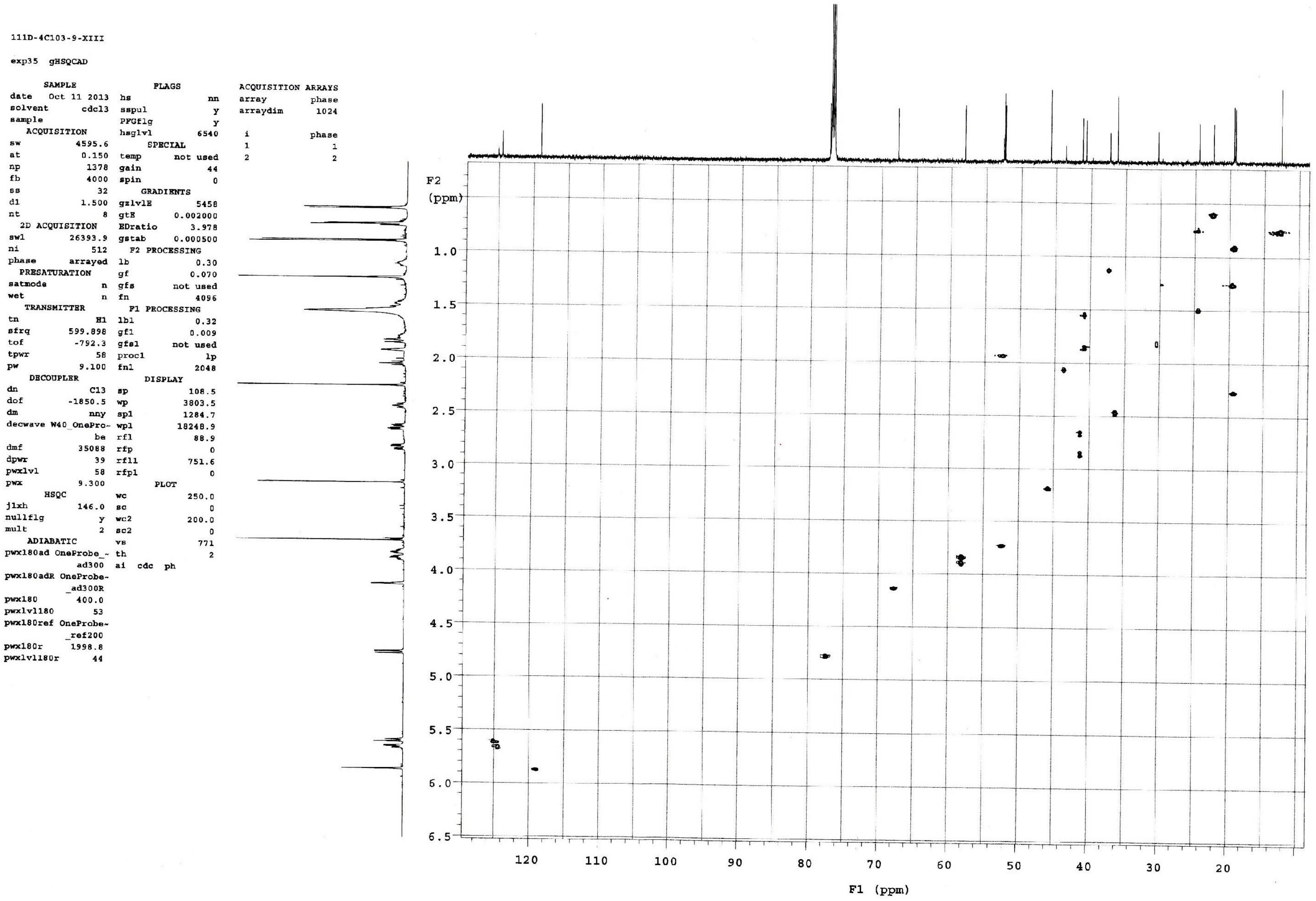

Figure S6. HSQC of 1.

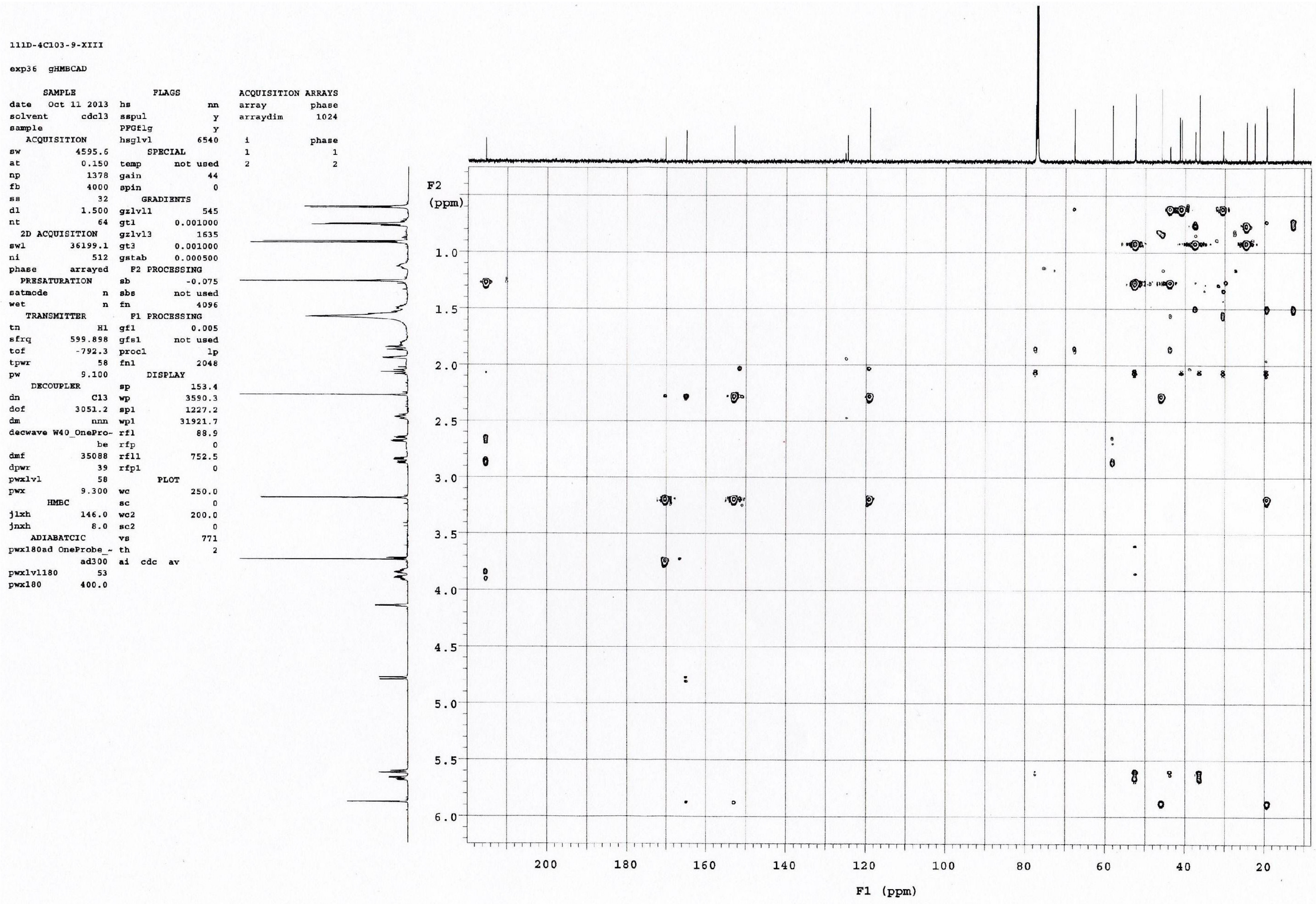

Figure S7. HMBC of 1.

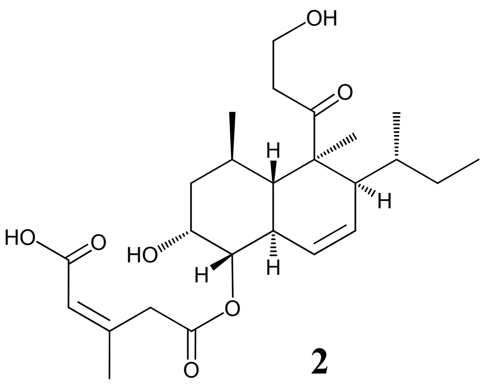

2

Figure S8. Compound 2.

Table S2. Spectral data including 2D NMR data for 2.

Position	δH [a]		J/Hz	¹H-¹H COSY	NOE [b]	δC		HMBC (C) [c]
1A	3.84	ddd	10.8 (2B), 6.5 (2A), 3.5 (2B)	1B, 2A, 2B		58.0	(t)	2, 3
1B	3.90	ddd	10.8 (1B), 7.5 (2B), 3.5 (2A)	1A, 2A, 2B	14			2, 3
2A	2.67	ddd	18.5 (2B), 6.5 (1A), 3.5 (1B)	1A, 1B, 2B	13, 14, 19	41.1	(t)	1, 3
2B	2.85	ddd	18.5 (1B), 7.5 (1B), 3.5 (1A)	1A, 1B, 2A	18, 19			1, 3
3						215.2	(s)	
4						52.5	(s)	
5	2.03	t	10.8 (6, 10)	6, 10	7β, 9, 15B, 18	43.4	(d)	3, 4, 6, 7, 9, 10, 13, 19
6	1.73	m		5, 7β, 18	10, 18, 19	30.5	(d)	
7α	1.83	dt	12.0 (7α), 3.0 (6, 8)	7β, 8	18	40.1	(t)	5, 8, 9
7β	1.53	td	12.0 (6, 7β), 3.0 (8)	6, 7α, 8	5, 9, 18			6
8	4.28	q	3.0 (7α, 7β, 9)	7α, 7β, 9		66.7	(d)	
9	4.55	dd	12.5 (10), 3.0 (8)	8, 10	5, 7β, 11	78.8	(d)	1′
10	2.47	br t	12.5 (5, 9)	5, 9	6, 19	36.0	(d)	5, 6, 9
11	5.69	dr d	10.8 (12)		9	125.1	(d)	5, 9, 10, 13
12	5.67	dd	10.8 (11), 4.2 (13)	13	16, 17	124.5	(d)	4, 10, 13
13	1.94	m		12, 14	2A, 17, 19	52.3	(d)	4, 5, 14, 15, 17, 19
14	1.12	m		13, 15A, 17	1B, 2A, 16	37.2	(t)	
15A	0.72	m		14, 15B, 17	17	24.5	(d)	14, 16, 17
15B	1.47	m		15A, 16	5			14, 16, 17
16	0.75	t	7.1 (15)	15B	12, 14, 17	12.5	(q)	14, 15
17	0.92	d	7.1 (14)	14	12, 13, 15A, 16	19.2	(q)	14, 15, 16
18	0.59	d	6.0 (6)	6	2B, 5, 7α, 7β	22.3	(q)	5, 6, 7
19	1.26	s			2A, 2B, 6, 10, 13	19.3	(q)	3, 4, 5, 13
1′						170.0	(s)	
2′A	3.29	d	15.2 (2′B)	2′B	6′	39.7	(t)	1′, 3′, 4′, 6′
2′B	3.77	d	15.2 (2′A)	2′A				1′, 3′, 4′, 6′
3′						153.4	(s)	
4′	5.94	s			6′	118.5	(d)	2′, 5′, 6′
5′						168.9	(s)	
6′	2.08	s			2′A, 4′	27.4	(q)	2′, 3′, 4′

[a] ¹H chemical shift values (δppm from $SiMe_4$) followed by multiplicity and then the coupling constants (*J*/Hz). Figures in parentheses indicate the proton coupling with that position; [b] The correlations with geminal and vicinal protones are removed; [c] Long range ¹H-¹³C correlations from H to C observed in the HMBC experiment.

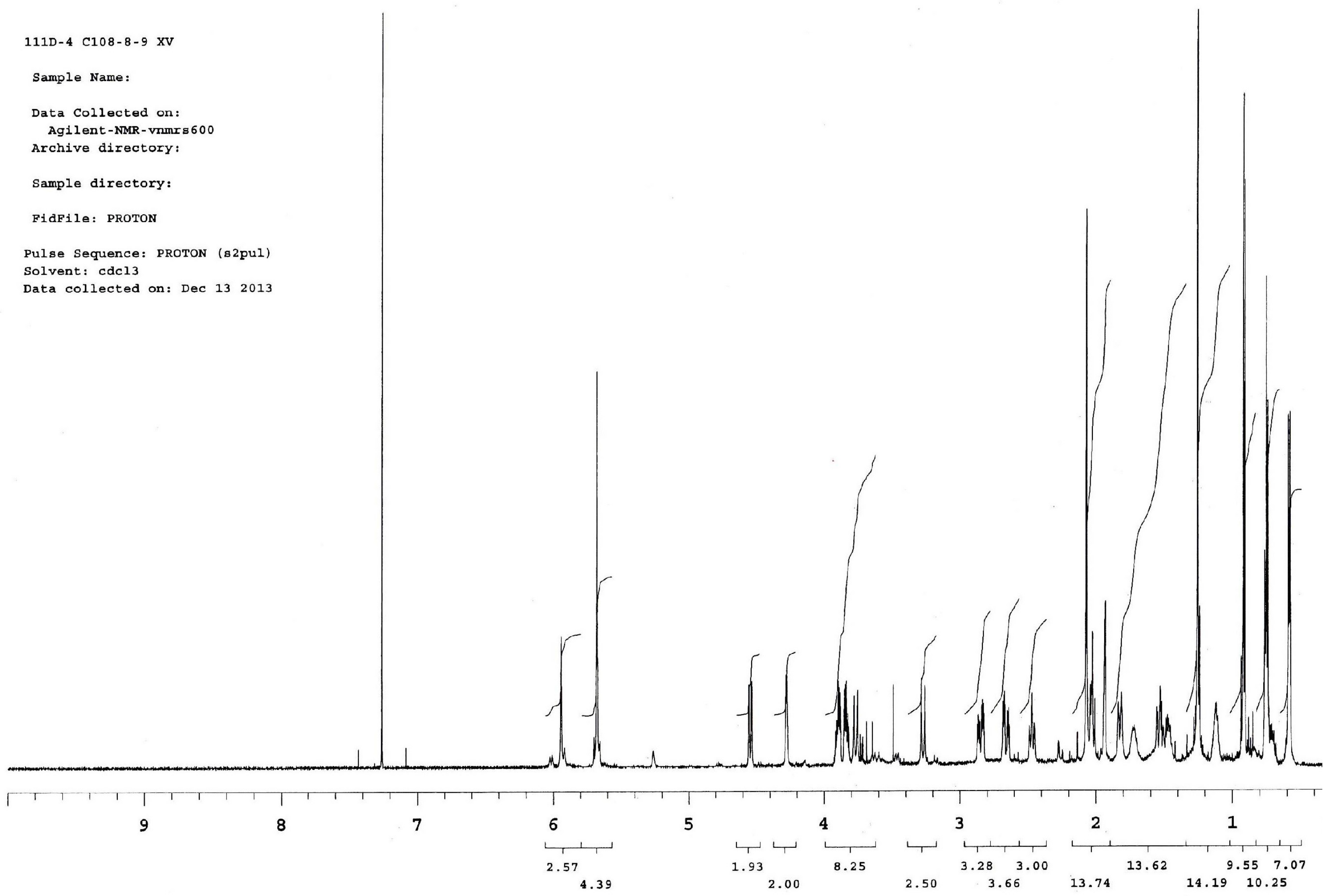

Figure S9. ^{1}H NMR spectrum of 2.

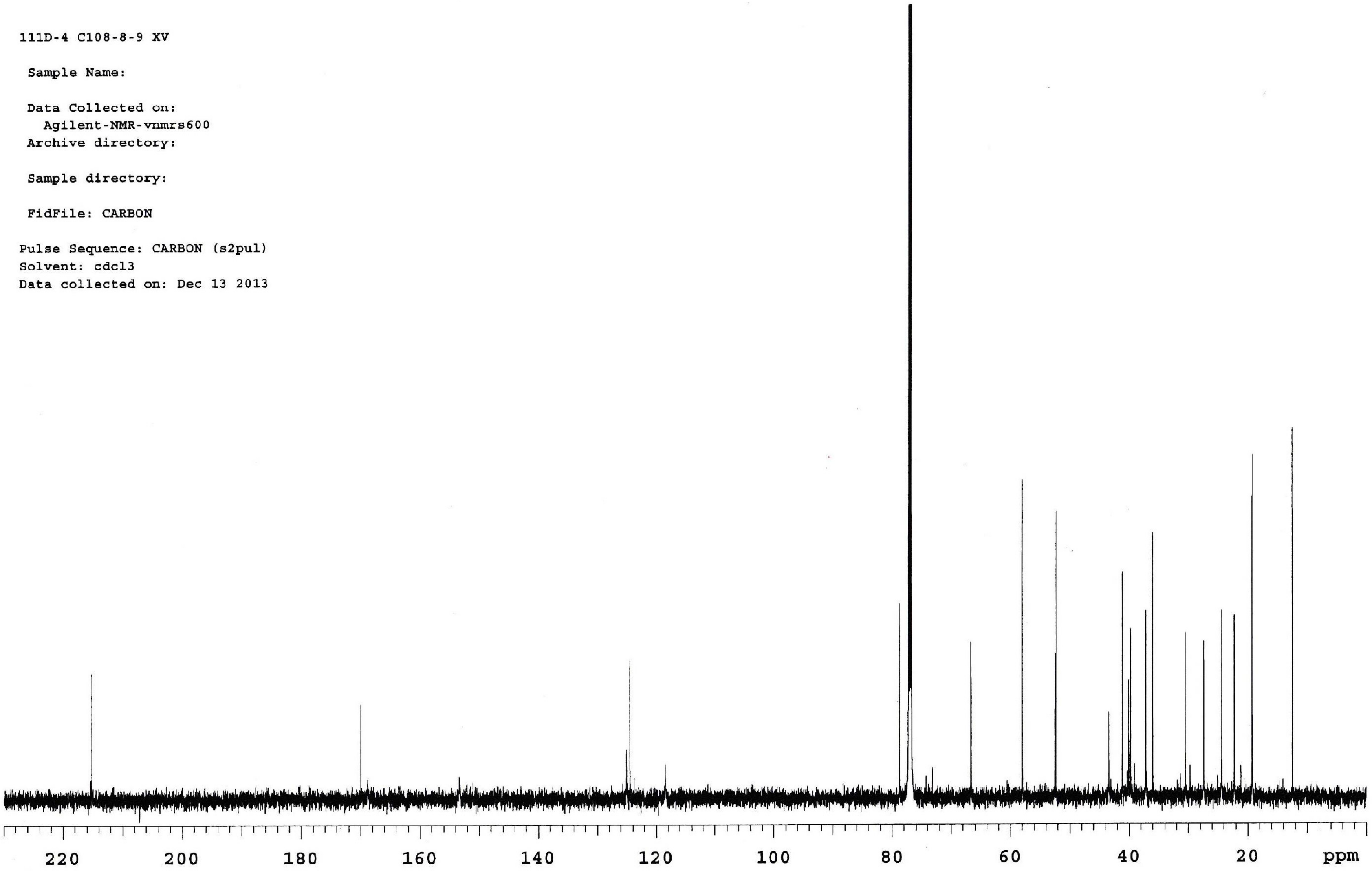

Figure S10. ^{13}C NMR spectrum of 2.

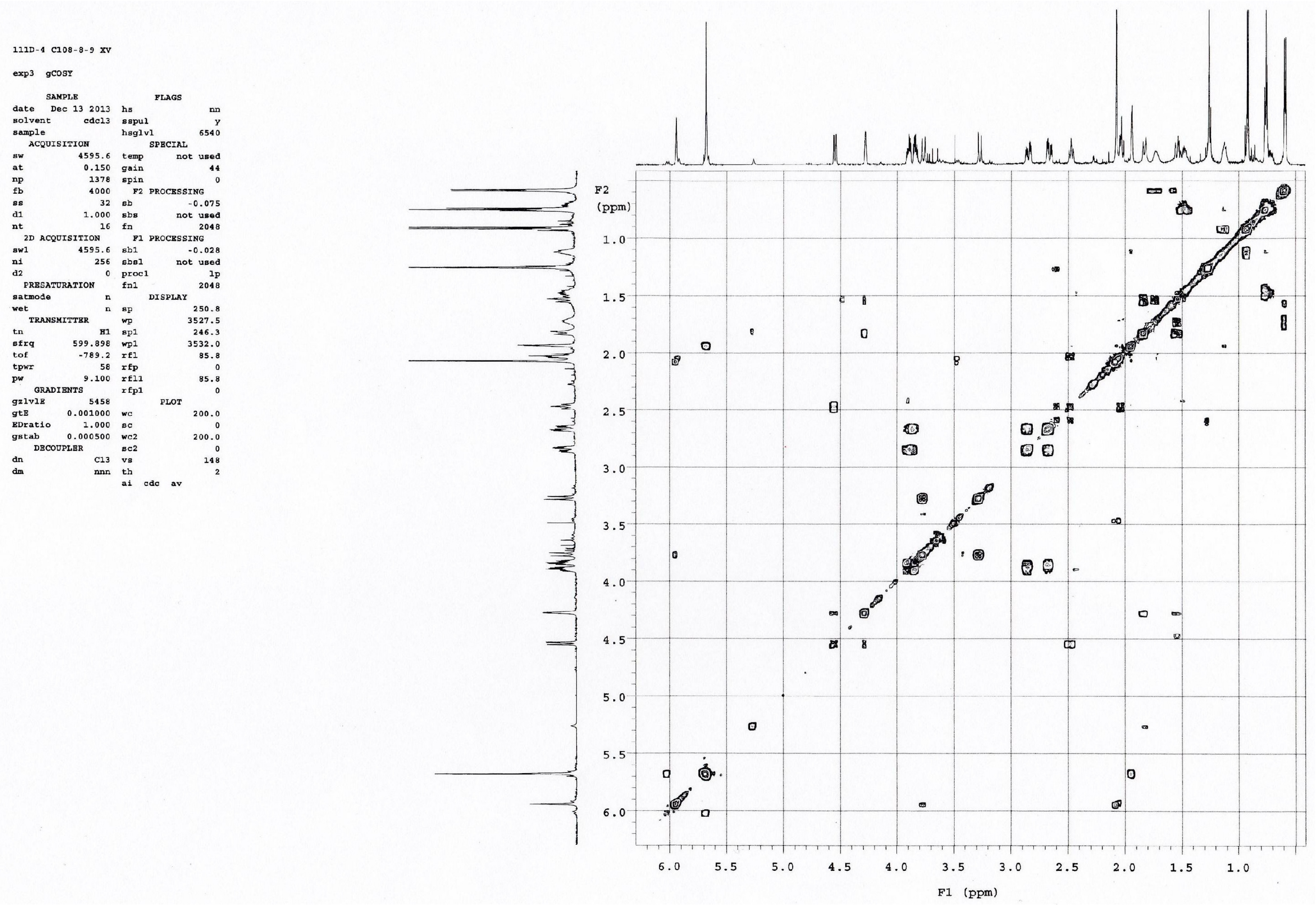

Figure S11. ^1H-^1H COSY of 2.

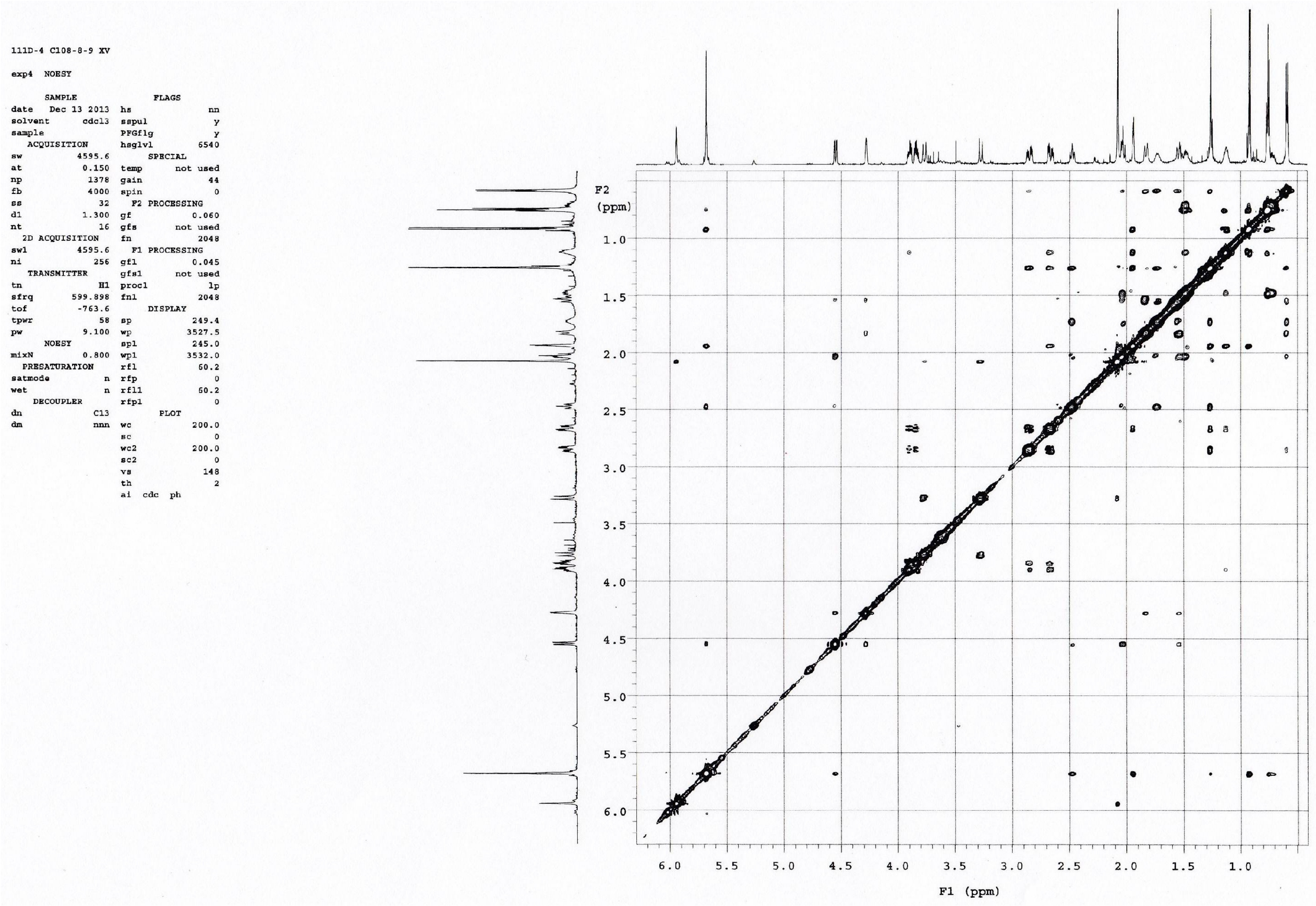

Figure S12. NOESY of 2.

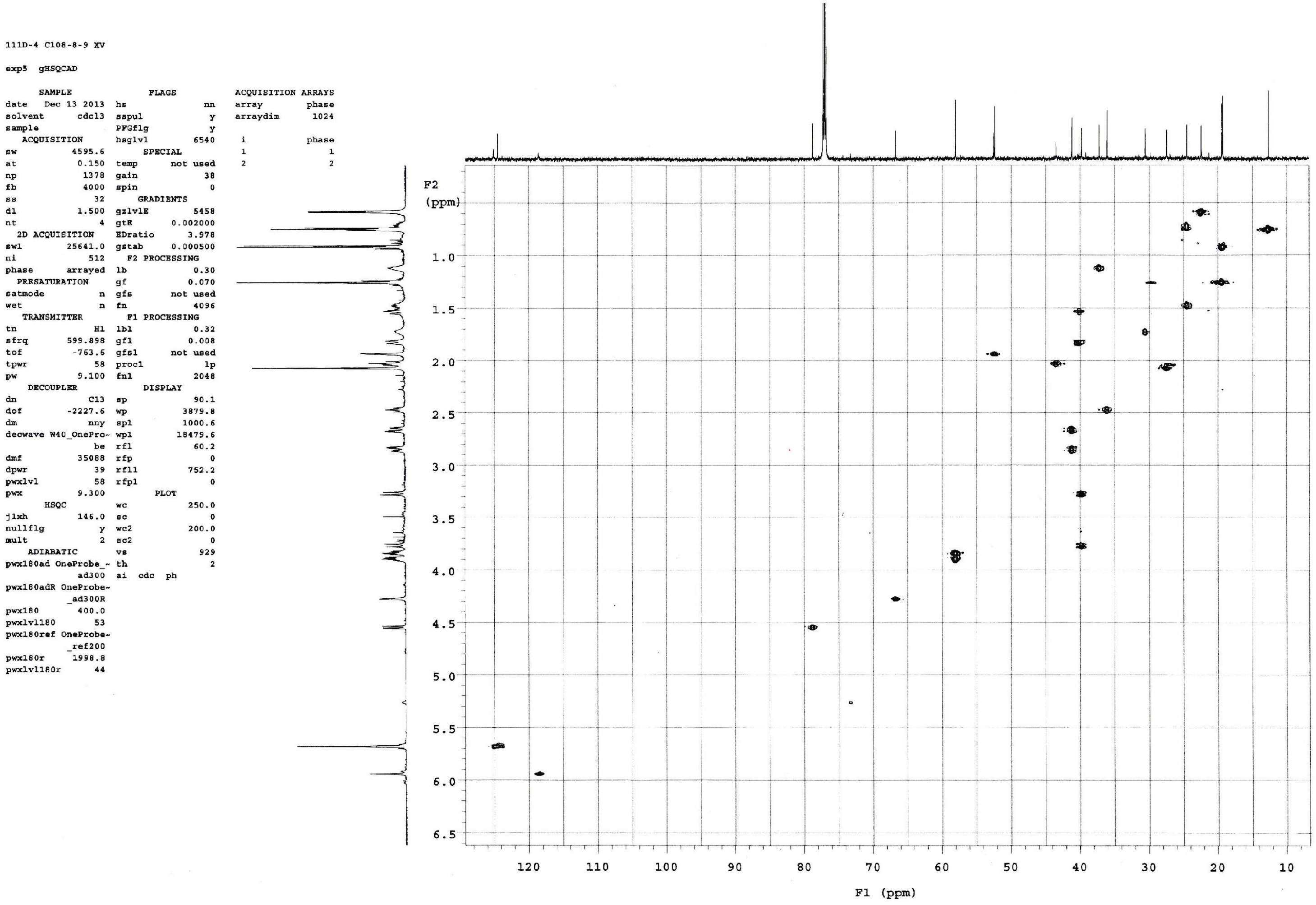

Figure S13. HSQC of 2.

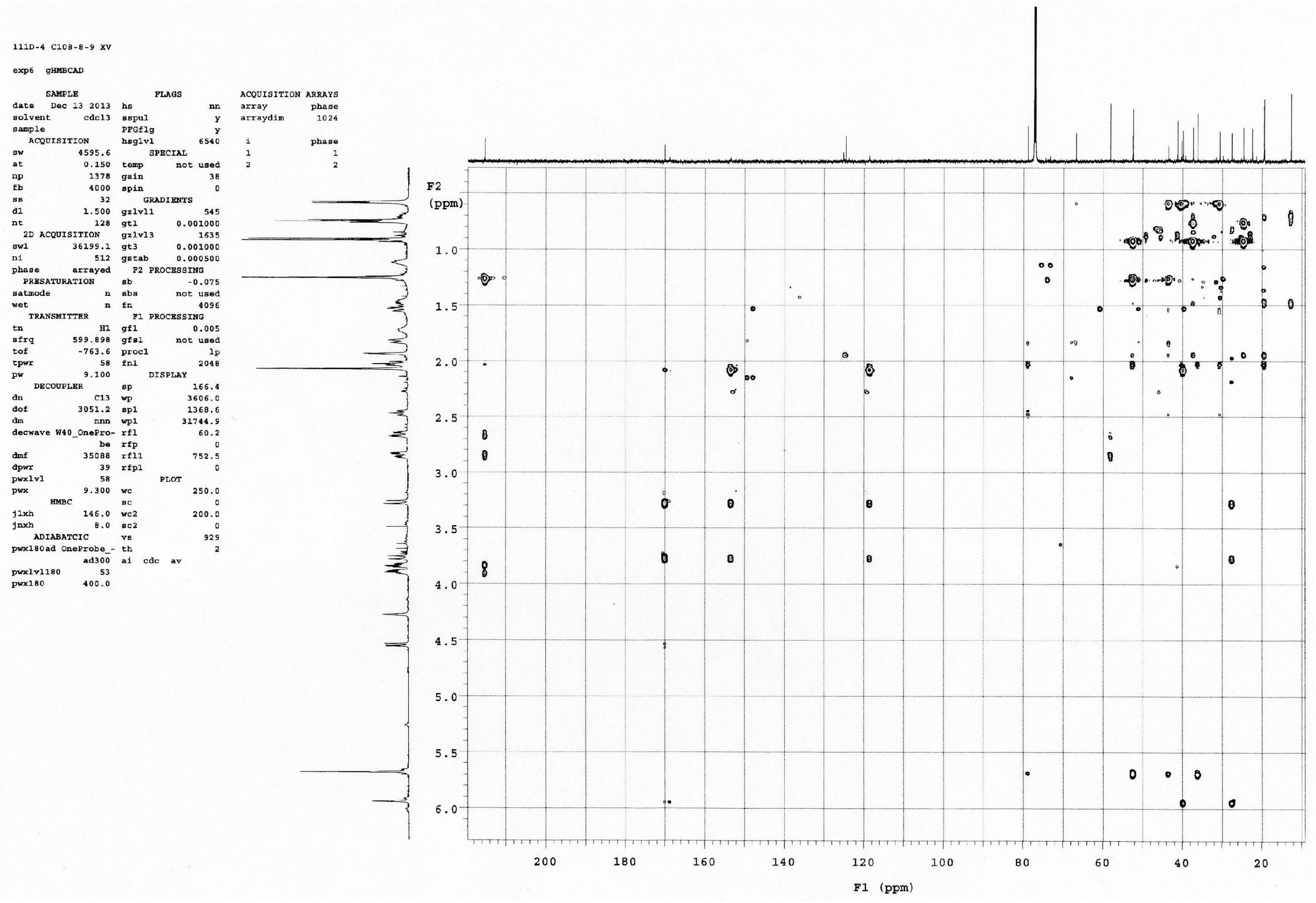

Figure S14. HMBC of 2.

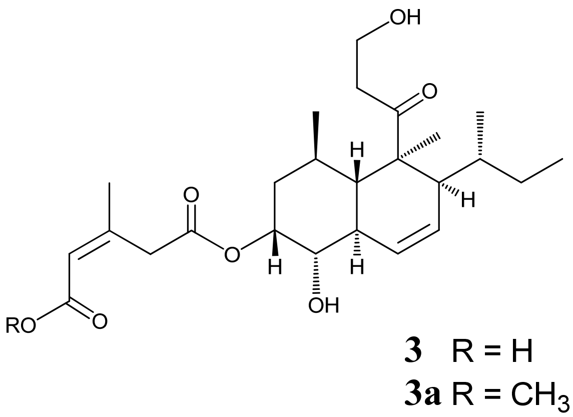

3 R = H
3a R = CH_3

Figure S15. Compound 3.

Table S3. Spectral data including 2D NMR data for 3.

Position	δ_H [a]		*J*/Hz	^{1}H-^{1}H COSY	NOE [b]	δ_C		HMBC (C) [c]
1A	3.83	ddd	11.8 (2B), 6.5 (2A), 3.5 (2B)	1B, 2A, 2B		58.0	(t)	3
1B	3.91	ddd	11.8 (1B), 6.5 (2B), 3.5 (2A)	1A, 2A, 2B	14			3
2A	2.67	ddd	18.5 (2B), 6.5 (1A), 3.5 (1B)	1A, 1B, 2B	13, 14, 19	41.2	(t)	1, 3
2B	2.86	ddd	18.5 (1B), 6.5 (1B), 3.5 (1A)	1A, 1B, 2A	18, 19			1, 3
3						215.2	(s)	
4						52.5	(s)	
5	1.96	t	10.8 (6, 10)	6, 10	7β, 9, 15B, 18	43.1	(d)	3, 4, 6, 7, 9, 10, 13, 19
6	1.59	m		5, 7β, 18	10, 19	31.4	(d)	18
7α	1.83	dt	12.0 (7α), 3.0 (6, 8)	7β, 8	18	39.1	(t)	5, 8, 9
7β	1.55	td	12.0 (6, 7β), 3.0 (8)	6, 7α, 8	5, 9, 18			6
8	5.26	q	3.0(7α, 7β, 9)	7α, 7β, 9		73.3	(d)	
9	3.48	dd	10.8 (10), 3.0 (8)	8, 10	5, 7β, 11	74.2	(d)	8, 10
10	2.08	tdd	10.8 (5, 9), 4.2 (11), 2.4 (12)	5	6, 19	40.4	(d)	9
11	6.06	dt	10.8 (12), 4.2 (11, 13)	12, 13	9	125.9	(d)	5, 9, 10, 13
12	5.69	ddd	10.8 (11), 4.2 (13), 2.4 (10)	11, 13	16, 17	123.7	(d)	10, 13, 14
13	1.94	m		11, 12, 14	2A, 17, 19	52.4	(d)	4, 12, 19
14	1.12	m		13, 15A, 17	1B, 2A, 16	37.2	(t)	
15A	0.74	m		14	17	24.4	(d)	16
15B	1.47	m		16	5			14, 16, 17
16	0.76	t	7.3 (15)	15B	12, 14, 17	12.5	(q)	14, 15
17	0.93	d	7.2 (14)	14	12, 13, 15A, 16	19.2	(q)	14, 15, 16
18	0.59	d	6.0 (6)	6	2B, 5, 7α, 7β	22.3	(q)	5, 6, 7
19	1.25	s			2A, 2B, 6, 10, 13	19.3	(q)	3, 4, 5, 13
1′						169.8	(s)	
2′A	3.54	d	15.2 (2′B)		6′	39.6	(t)	1′, 3′, 4′, 6′
2′B	3.73	d	15.2 (2′A)		6′			1′, 3′, 4′, 6′
3′						153.9	(s)	
4	5.92	s			6′	118.1	(d)	2′, 5′, 6′
5′						168.9	(s)	
6′	2.05	s			2′A, 2′B, 4′	26.9	(q)	2′, 3′, 4′

[a] ^{1}H chemical shift values (δppm from $SiMe_4$) followed by multiplicity and then the coupling constants (*J*/Hz). Figures in parentheses indicate the proton coupling with that position; [b] The correlations with geminal and vicinal protones are removed; [c] Long range ^{1}H-^{13}C correlations from H to C observed in the HMBC experiment.

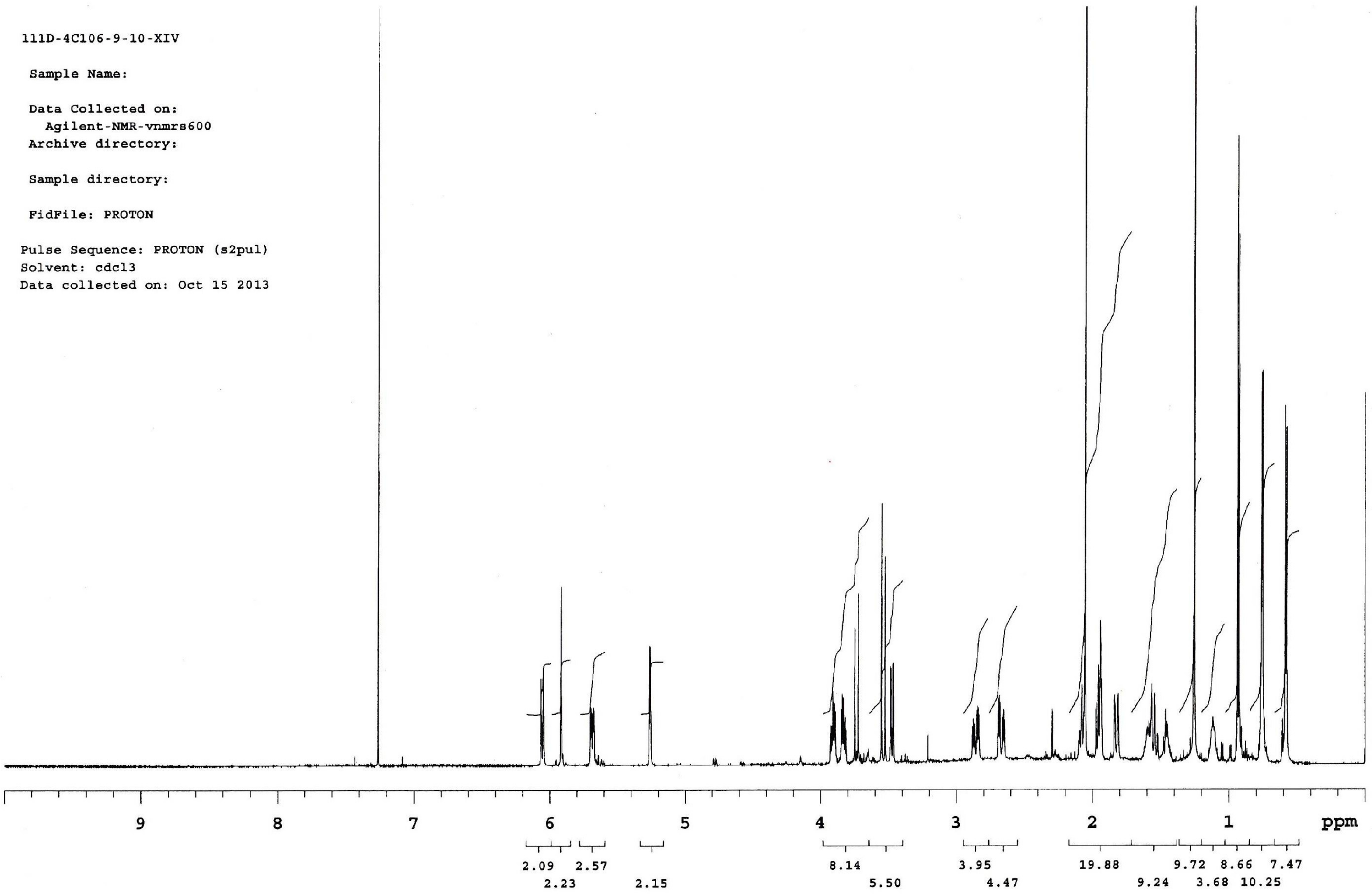

Figure S16. ^{1}H NMR spectrum of 3.

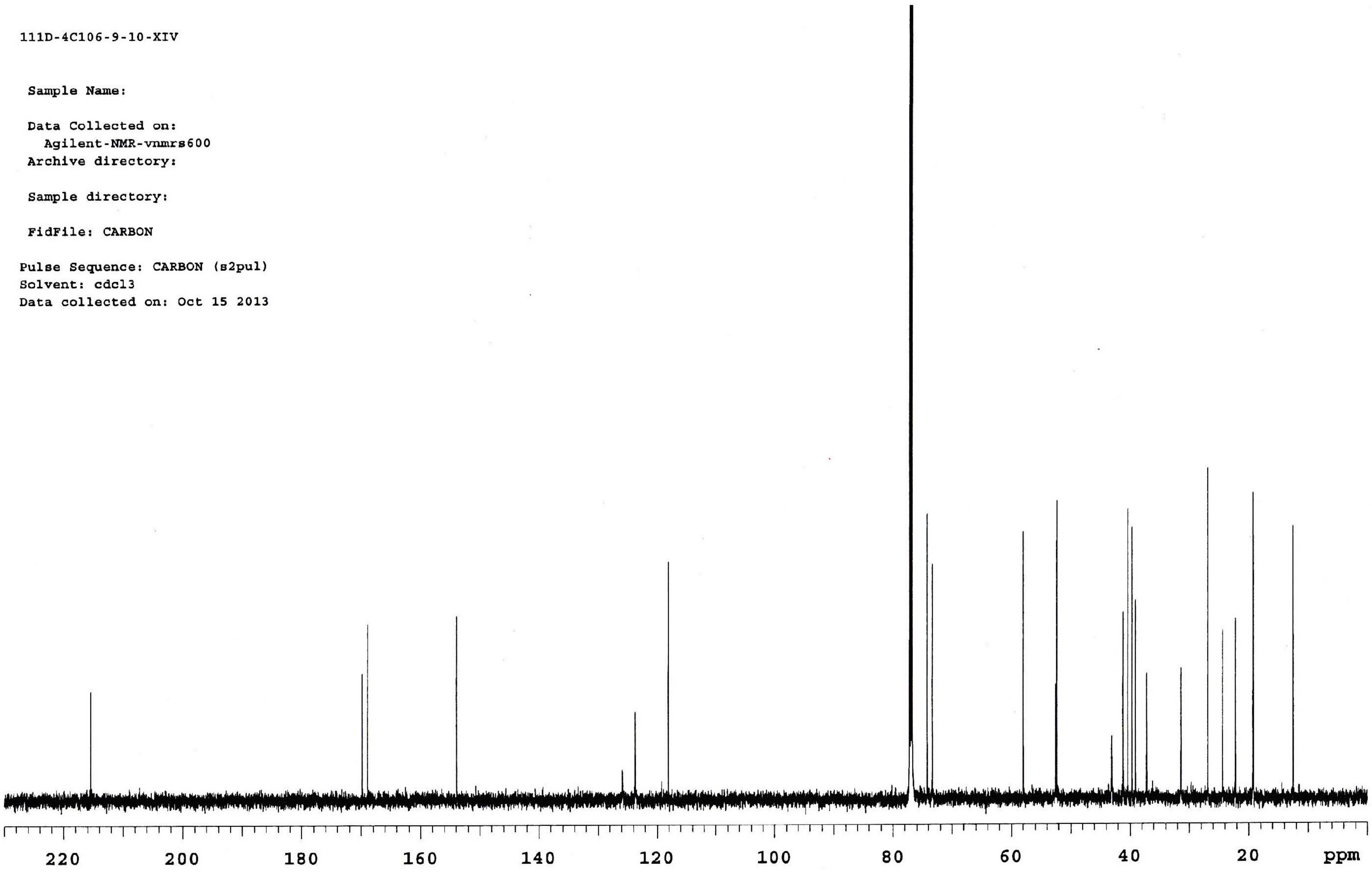

Figure S17. ^{13}C NMR spectrum of 3.

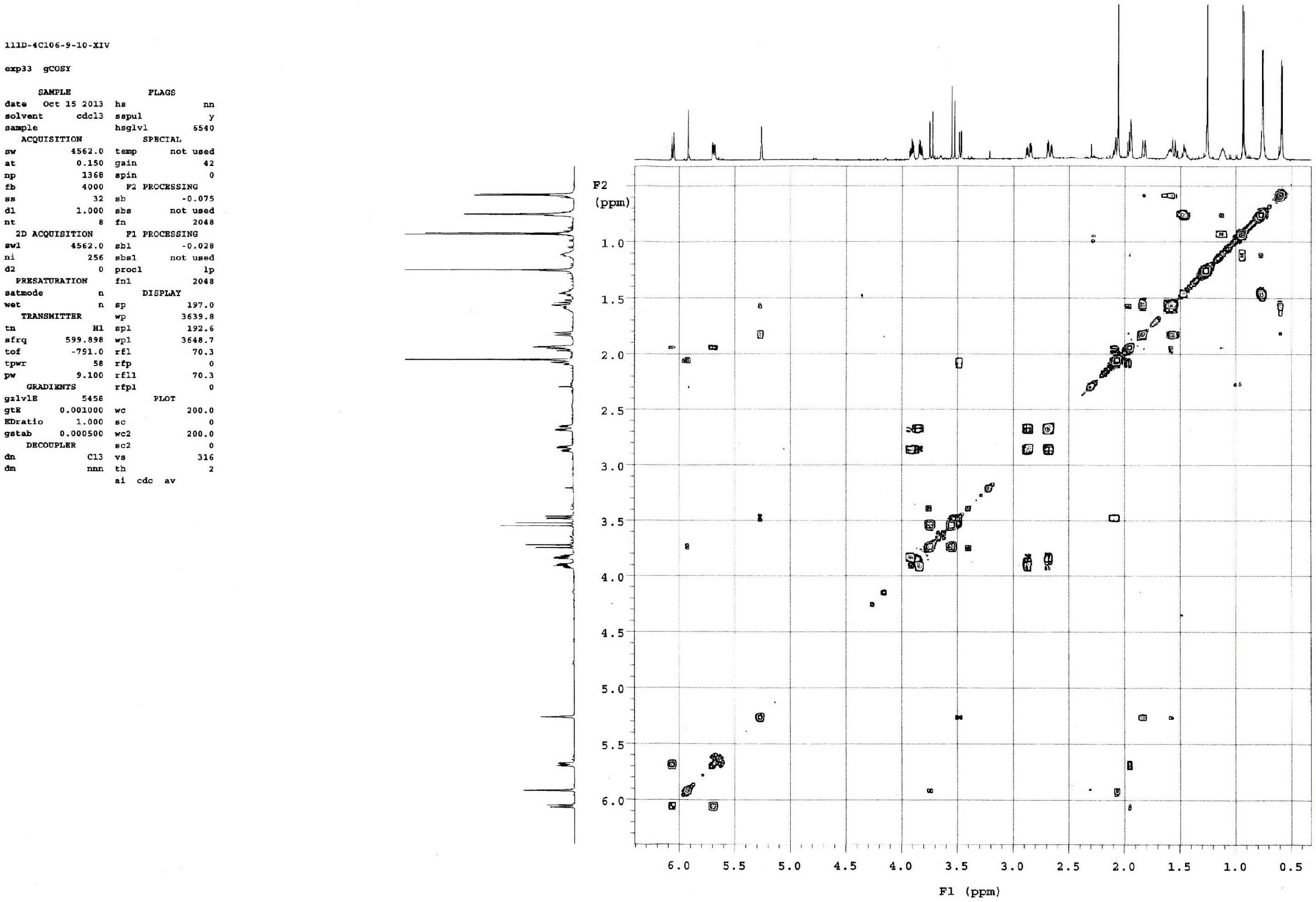

Figure S18. ^{1}H-^{1}H COSY of 3.

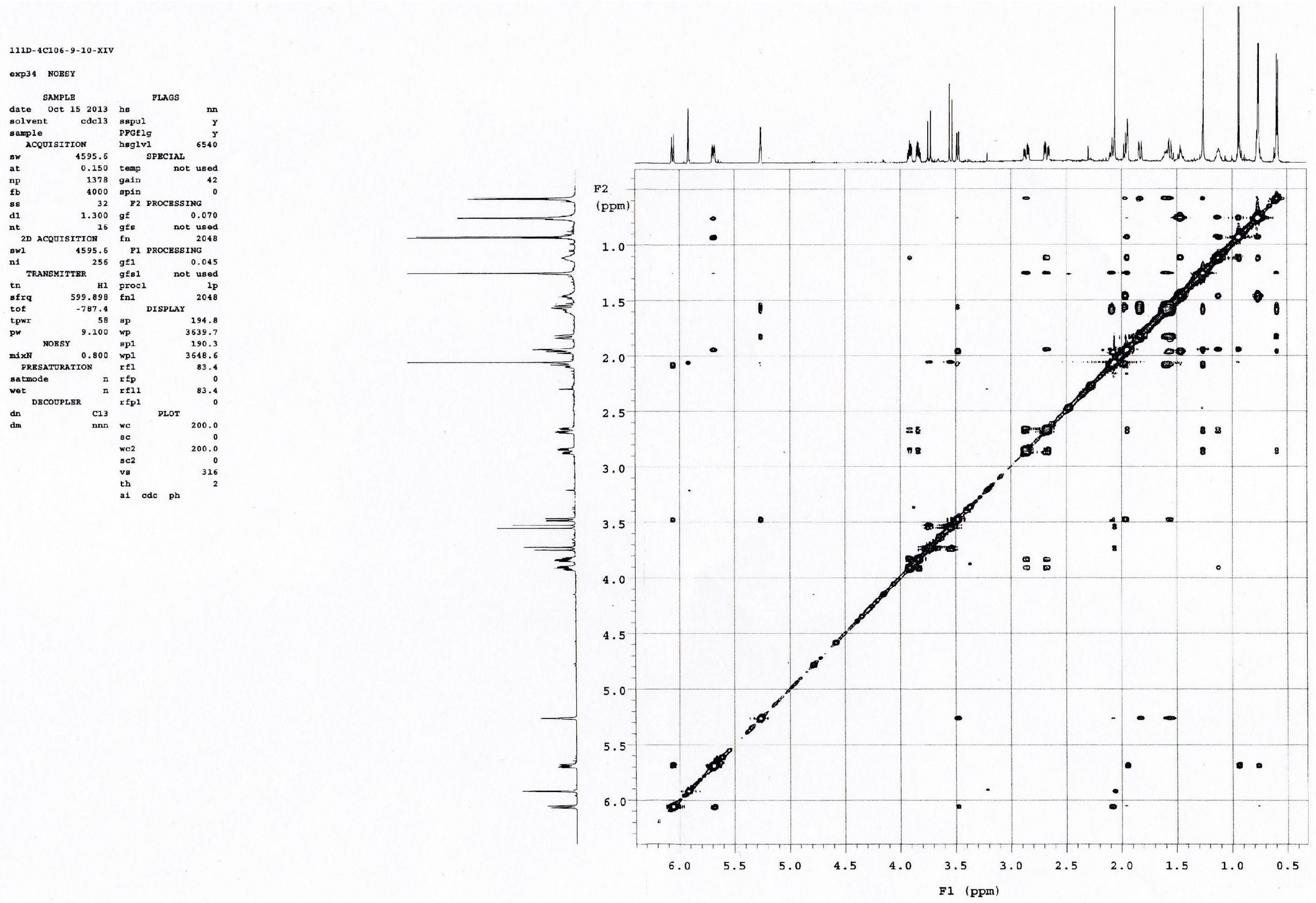

Figure S19. NOESY of 3.

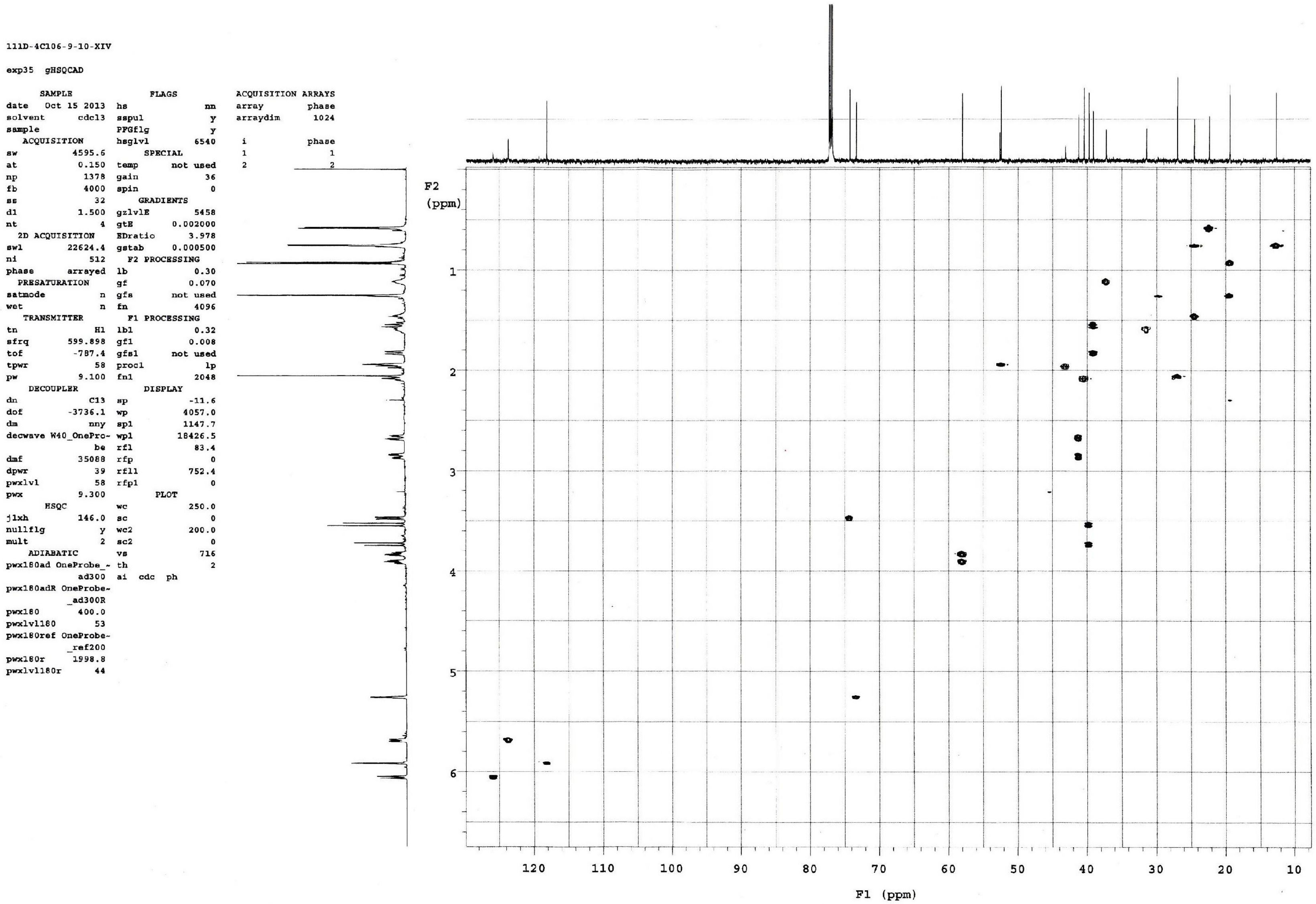

Figure S20. HSQC of 3.

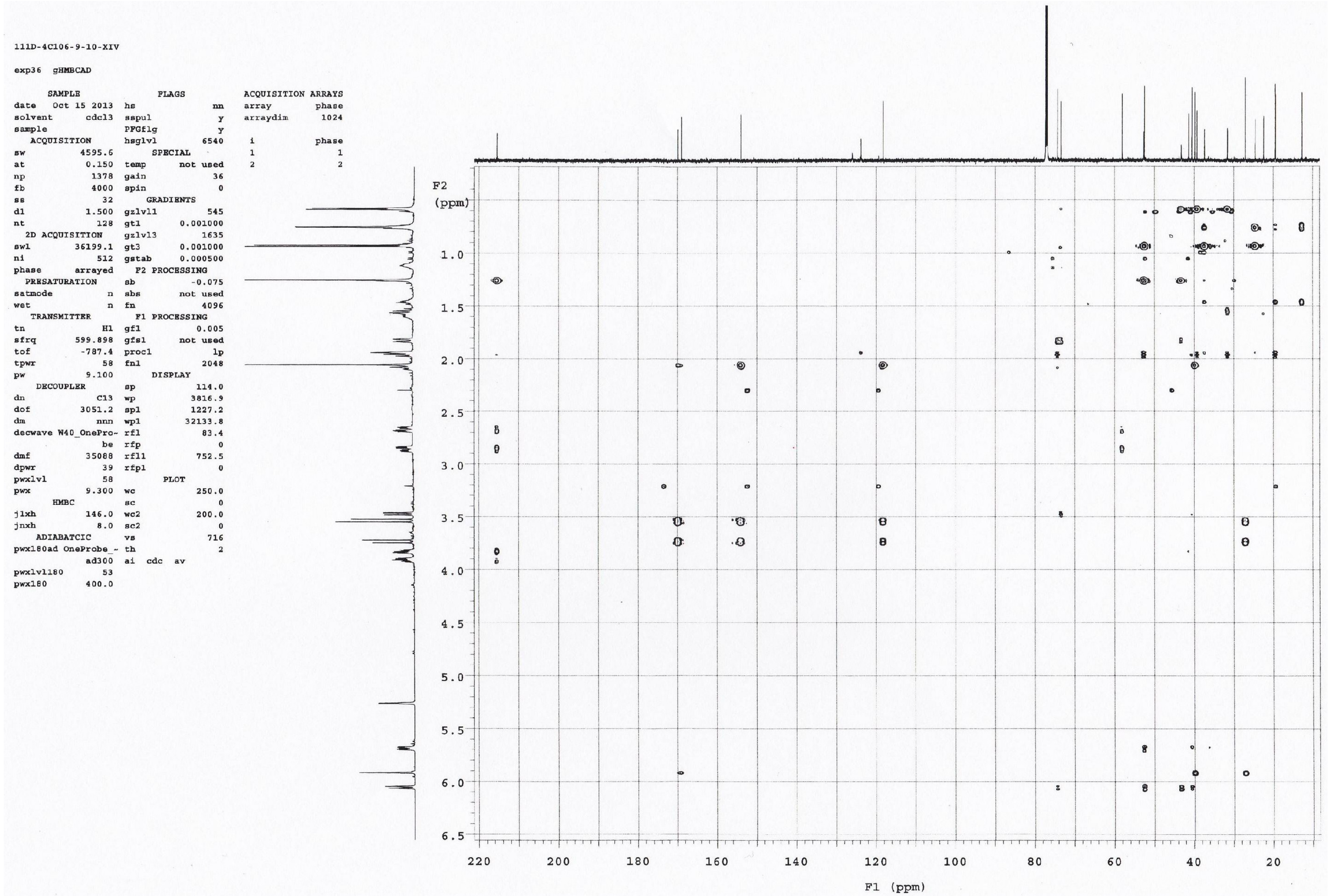

Figure S21. HMBC of 3.

Table S4. Spectral data including 2D NMR data for 3a.

Position	δ_H [a]		*J*/Hz	^{1}H-^{1}H COSY	NOE [b]	δ_C		HMBC (C) [c]
1A	3.84	m		1B, 2A, 2B		58.0	(t)	
1B	3.90	m		1A, 2A, 2B				
2A	2.67	ddd	18.5 (2B), 6.0 (1A), 3.0 (1B)	1A, 1B, 2B	13, 14, 19	41.2	(t)	3
2B	2.85	ddd	18.5 (1B), 6.0 (1B), 3.0 (1A)	1A, 1B, 2A	18, 19			1, 3
3						215.5	(s)	
4						52.6	(s)	
5	1.96	t	10.2 (6, 10)	6, 10	7β, 9, 15B, 18	43.1	(d)	4, 6, 7, 9, 10, 19
6	1.60	m		5, 7α, 18		31.4	(d)	
7α	1.84	dt	12.0 (7α), 3.0 (6, 8)	6, 7β, 8	18	39.1	(t)	9
7β	1.58	m		7α, 8	5, 9, 18			6
8	5.26	q	3.0 (7α, 7β, 9)	7α, 7β, 9		73.5	(d)	
9	3.46	m		8, 10	5, 7β, 11	74.2	(d)	
10	2.06	m		5	19	40.5	(d)	
11	6.09	m		12	9	126.0	(d)	5, 13
12	5.69	m		11, 13	16, 17	123.6	(d)	
13	1.94	m		12	2A, 17, 19	52.4	(d)	5, 19
14	1.12	m		15A, 17	2A, 16	37.2	(t)	
15A	0.76	m		14		24.4	(d)	
15B	1.46	m		16	5			16, 17
16	0.76	t	7.3 (15)	15B	12, 14, 17	12.5	(q)	14, 15
17	0.93	d	7.2 (14)	14	12, 13, 16	19.2	(q)	14, 15, 16
18	0.59	d	6.0 (6)	6	2B, 5, 7α, 7β	22.3	(q)	5, 6, 7
19	1.26	s			2A, 2B, 10, 13	19.3	(q)	3, 4, 5, 13
1′						170.0	(s)	
2′A	3.60	d	15.2 (2′B)	2′B	6′	39.5	(t)	1′, 3′, 4′, 6′
2′B	3.72	d	15.2 (2′A)	2′A	6′			1′, 3′, 4′, 6′
3′						152.1	(s)	
4	5.88	s			6′	118.4	(d)	2′, 5′, 6′
5′						167.0	(s)	
6′	2.03	s			2′A, 2′B, 4′	26.6	(q)	2′, 3′, 4′
5′-OCH$_3$		3.68	s				51.3	(q), 5′

[a] ^{1}H chemical shift values (δppm from $SiMe_4$) followed by multiplicity and then the coupling constants (*J*/Hz). Figures in parentheses indicate the proton coupling with that position; [b] The correlations with geminal and vicinal protones are removed; [c] Long range ^{1}H-^{13}C correlations from H to C observed in the HMBC experiment.

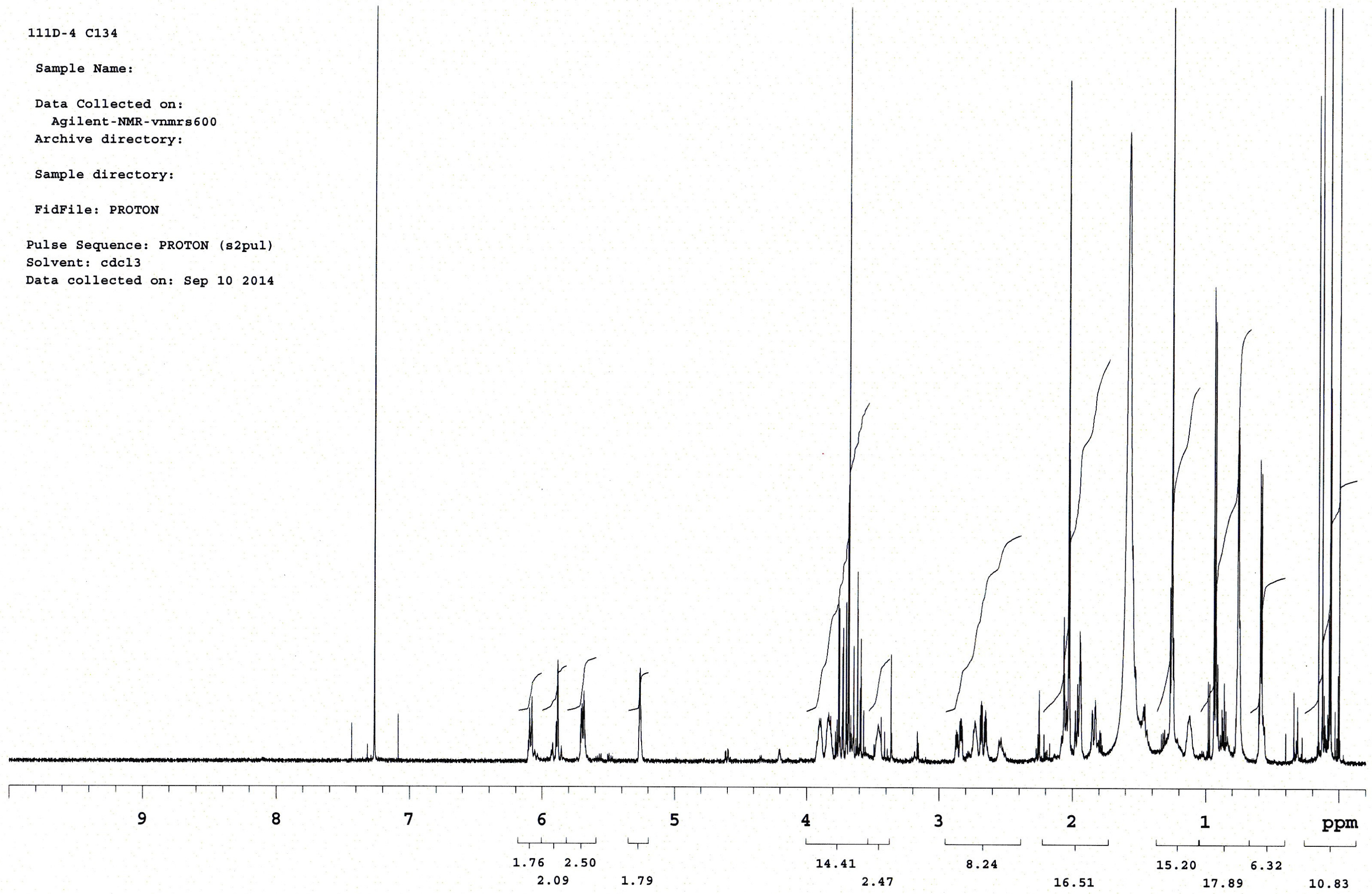

Figure S22. ^{1}H NMR spectrum of 3a.

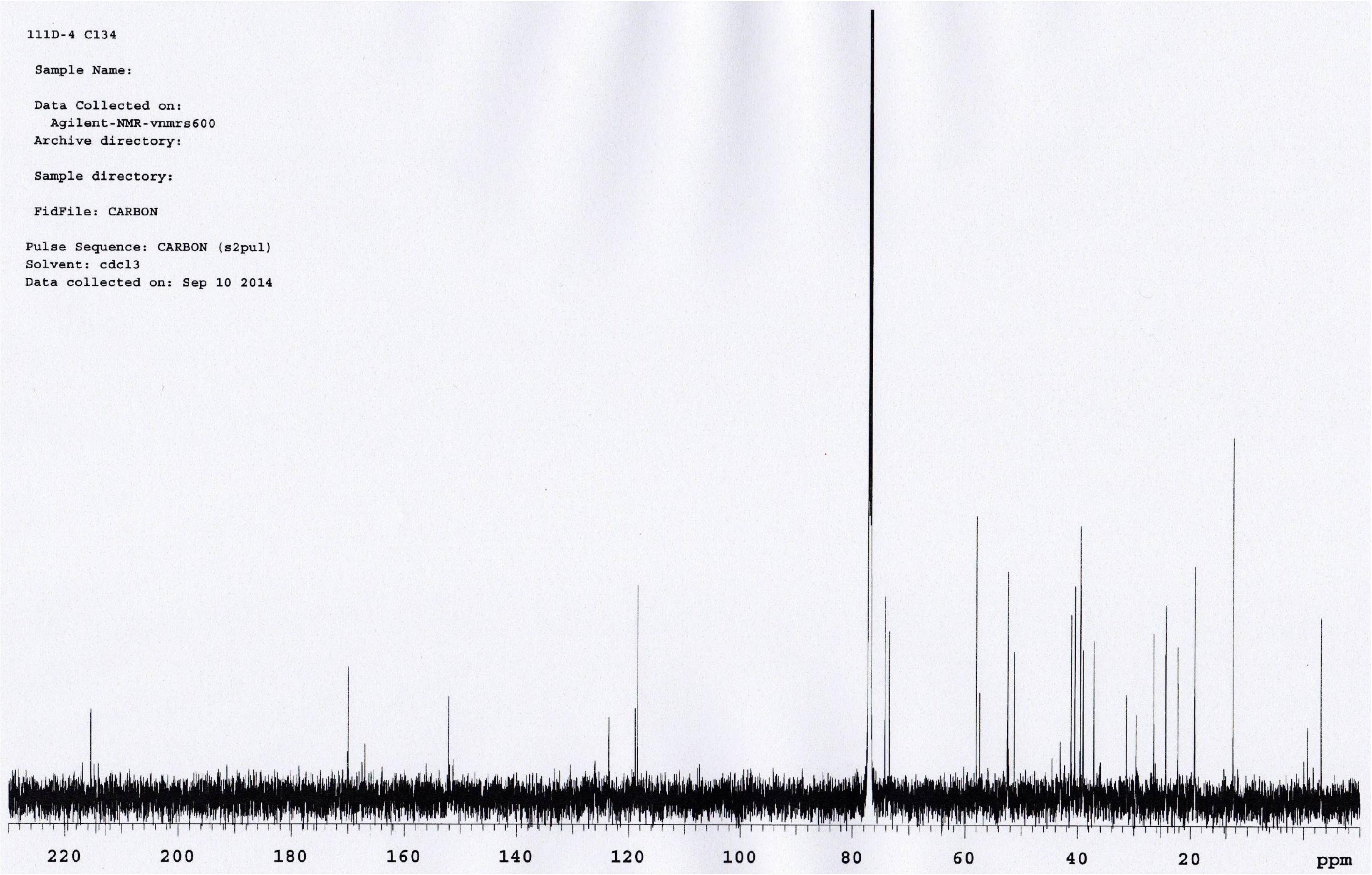

Figure S23. ^{13}C NMR spectrum of 3a.

© 2015 by the authors; licensee MDPI, Basel, Switzerland. This article is an open access article distributed under the terms and conditions of the Creative Commons Attribution license (http://creativecommons.org/licenses/by/4.0/).
